# Purification of Alkaloids from *Zanthoxylum bungeanum* Using Macroporous Adsorption Resin and Evaluation of Their Biological Activities

**DOI:** 10.3390/molecules31132328

**Published:** 2026-07-02

**Authors:** Dongdong Huang, Tianyu Zhao, Bohao Wang, Benqun Yang, Zhifeng Li

**Affiliations:** 1 College of Chemical Engineering and Technology, Tianshui Normal University, Tianshui 741001, China; ddhtsnu@126.com (D.H.); zhaoty08@126.com (T.Z.); 15294360059@163.com (B.W.); 2 Key Laboratory of Advanced Optoelectronic Functional Materials of Gansu Province, Tianshui Normal University, Tianshui 741001, China; 3 Key Laboratory for New Molecule Materials Design and Function of Gansu Universities, Tianshui Normal University, Tianshui 741001, China

**Keywords:** *Zanthoxylum bungeanum*, alkaloids, antioxidant, antibacterial

## Abstract

*Zanthoxylum bungeanum* alkaloids have attracted considerable attention for their potential health benefits, yet systematic investigations into their extraction, purification, and comprehensive bioactivity remain scarce. In this study, an efficient extraction protocol was developed and scaled up, followed by purification using macroporous resin NKA-9 with 70% ethanol as the optimal eluent. Ultra-performance liquid chromatography coupled with ion mobility quadrupole time-of-flight mass spectrometry (UPLC-IM-QTOF/MS) identified 11 major alkaloids in the 70% ethanol eluate fraction. The antioxidant capacity of the 70% ethanol eluate fraction was evaluated through DPPH, ABTS^+^·, and Fe^3+^ reducing power assays, revealing a clear dose-dependent effect, albeit weaker than ascorbic acid. Antibacterial screening against four pathogenic bacteria (*Escherichia coli*, *Bacillus subtilis*, *Staphylococcus aureus*, and *Pseudomonas aeruginosa*) via the Oxford cup method, macrobroth dilution, and minimum bactericidal concentration (MBC) determination demonstrated strain-selective activity. The strongest effect was observed against *E. coli* [minimum inhibitory concentration (MIC) = 2 mg/mL, MBC = 8 mg/mL, MBC/MIC = 4, bactericidal], followed by moderate activity against *B. subtilis* (MIC = 4 mg/mL, MBC = 16 mg/mL), while only bacteriostatic effects were noted against *S. aureus* and *P. aeruginosa* within the tested range. These findings provide a robust foundation for further development of *Zanthoxylum bungeanum* alkaloids as natural functional ingredients or food-compatible bio preservatives.

## 1. Introduction

*Zanthoxylum bungeanum* Maxim., an aromatic species of the genus *Zanthoxylum* in the Rutaceae family, is well recognized for its distinctive fragrance and various bioactivities [1,2,3]. It has a long history of cultivation in China and is now extensively grown. As a traditional medicinal and edible plant, it is mainly distributed in southwest China [4]. Modern phytochemical studies have revealed that *Zanthoxylum bungeanum* exhibits a variety of pharmacological activities, such as analgesic, anti-inflammatory, antibacterial, anti-tumor, and antioxidant effects [5]. These active compounds include volatile oils [6,7], phenolic compounds (e.g., flavonoids) [1], alkaloids from related *Zanthoxylum* species [8], and ketones [9], among others. Over 140 compounds have been reported to be isolated and identified from the stems, leaves, seeds, and peels of *Zanthoxylum bungeanum* [10]. Among these, alkylamides (e.g., hydroxy-α-sanshool, HAS) and other alkaloids are the main characteristic components, exhibiting complex chemical profiles, diverse structures, and multiple pharmacological activities [11,12].

In recent years, there has been growing interest among both industry stakeholders and consumers in the potential nutritional value of *Zanthoxylum bungeanum* extracts. These extracts exhibit marked antioxidant capacity, effectively scavenging free radicals and thereby contributing to the prevention and potential management of chronic diseases [13]. Although flavonoids (typical phenolic compounds) have been extensively studied for their antioxidant properties, the antioxidant activity of plant-derived alkaloids remains relatively underexplored. However, previous studies have demonstrated that certain alkaloids possess notable biological activities [14]. For instance, specific compounds derived from plants have demonstrated significant antioxidant and anti-inflammatory effects in vitro, showing potential for disease treatment and prevention [15,16]. Therefore, the abundance and antioxidant potential of alkaloids have attracted significant research interest, establishing them as one of the most investigated classes of bioactive compounds. They are commonly applied in the pharmaceutical, functional food, and cosmetic industries, where they are increasingly utilized in the development of health-promoting products.

Extraction is the primary step in isolating bioactive compounds from plants. However, the efficiency of this process is significantly influenced by extraction temperature [17], type of solvent [18], and extraction techniques [19]. The development of green extraction technologies—such as ultrasound, microwave-assisted, and enzymatic extraction—has improved the yield of target compounds while minimizing detrimental effects on their biological activity. Previous studies [2] reported the use of response surface methodology (RSM) to optimize the extraction of *Zanthoxylum bungeanum* polysaccharides and to evaluate their bioactivity. However, research on the extraction technology of *Zanthoxylum bungeanum* alkaloids remains limited. Specifically, systematic comparisons of different macroporous resins for the enrichment of *Zanthoxylum bungeanum* total alkaloids under strictly identical conditions are lacking, and the quantitative effects of resin polarity on both purity and marker-amide recovery has not been fully clarified.

To address this, we present a comparative evaluation of NKA-9, D101, and AB-8 macroporous resins for the purification of *Zanthoxylum bungeanum* total alkaloids under carefully controlled static and dynamic conditions. While several previous studies have investigated the use of macroporous resins for alkaloid enrichment, they often focused on either antioxidant or antibacterial activities separately, with relatively few assessing both aspects concurrently. Our assessment simultaneously evaluates total alkaloid content and impurity removal, thereby offering a more comprehensive perspective on resin selection. This protocol may serve as a reproducible and scalable approach for downstream compositional analysis and bioassay-oriented quality control.

Therefore, in this study, we aimed to optimize the extraction conditions of *Zanthoxylum bungeanum* alkaloids and evaluate their antioxidant and antibacterial activities. First, we optimized the extraction parameters through single-factor experiments and orthogonal design to maximize yield. Second, we purified the alkaloid extracts using macroporous adsorption resins and analyzed their purification performance. Third, we evaluated the biological activities of the purified samples using in vitro antioxidant assays (DPPH, ABTS, and reducing power) and antibacterial tests. These findings are expected to provide a solid foundation for further functional development and mechanism research of *Zanthoxylum bungeanum* alkaloids.

## 2. Results and Discussion

### 2.1. Optimization of Zanthoxylum bungeanum Total Alkaloid Extraction Process

**Figure 1:**
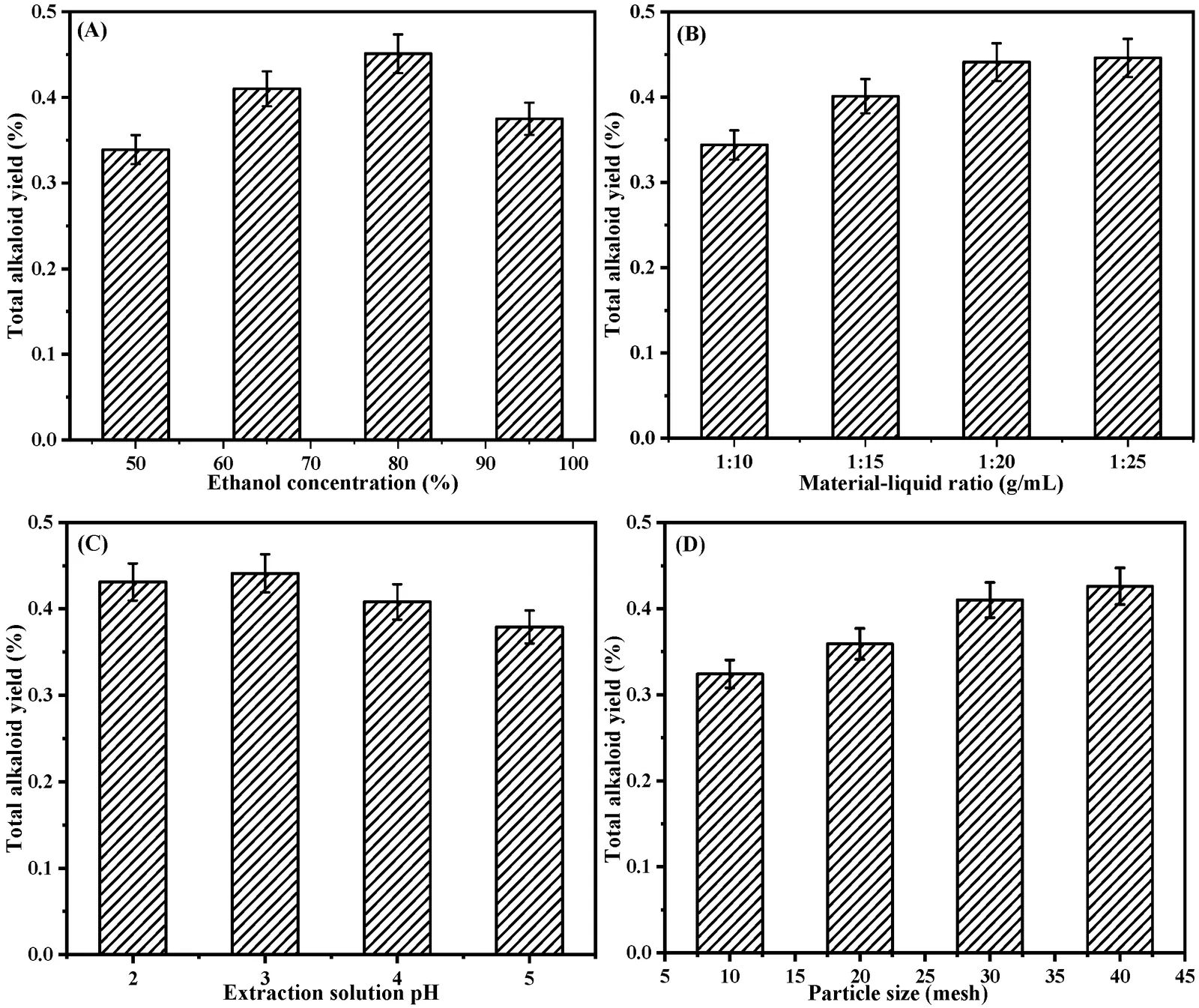
Effects of various extraction parameters on the total alkaloid yield. (**A**) ethanol concentration; (**B**) material-liquid ratio; (**C**) extraction solution pH; (**D**) particle size.

The yield of total alkaloids from *Zanthoxylum bungeanum* was significantly influenced by several key extraction parameters (Figure 1).

Effect of ethanol concentration: Ethanol, a versatile solvent capable of dissolving compounds across a wide polarity range, is effective for alkaloid extraction. Ethanol–water is a commonly used solvent system for alkaloid extraction [20,21]. As shown in Figure 1A, the total alkaloid yield initially increased and then decreased with increasing ethanol concentration. The maximum yield was achieved at 80% ethanol. However, further increasing the ethanol concentration to 95% reduced the yield to 0.375%. This trend indicates that a moderate proportion of water enhances the solubility of alkaloids.

Solid–liquid ratio: This parameter is a critical factor affecting extraction efficiency. Adjusting the solid–liquid ratio allows for precise control over the extraction of active compounds from plant materials. It influences both the leaching driving force and the efficiency of equilibrium mass transfer [22,23]. Generally, increasing the solid–liquid ratio facilitates alkaloid extraction. As shown in Figure 1B, with 15 g of *Zanthoxylum bungeanum*, the total alkaloid yield increased markedly as the solid–liquid ratio increased from 1:10 to 1:20. Beyond 1:20, the increase slowed. Previous studies have indicated that beyond a certain threshold (where the solvent volume is sufficient to ensure complete solvation and maintain a favorable mass-transfer driving force), further increases in volume lead to diminishing returns, merely increasing solvent waste and the energy required for subsequent evaporation [22,23].

Effect of pH on extraction: Adjusting the pH of the extraction solution can alter the dissociation state of the target compounds, thereby affecting extraction efficiency [24]. Since most alkaloids in *Zanthoxylum bungeanum* are basic, they readily form salts in acidic media, which improves their water solubility. When the pH was increased from pH 2 to pH 3, the total alkaloid yield increased accordingly, reaching its maximum at pH 3 (Figure 1C). These results suggest that an acidic environment promotes the dissolution of alkaloids in their salt form, thereby enhancing solubility. However, excessively acidic conditions may lead to the co-extraction of impurities or the destabilization of alkaloid structures.

Particle size: Figure 1D illustrates the influence of particle size on the total alkaloid yield. The results indicated that smaller particle sizes led to higher extraction yields. The yield increased as the particle size was reduced from 10-mesh to 40-mesh, indicating that finer particles enhance extraction efficiency. Reducing the particle size markedly increases the specific surface area, facilitating the diffusion of solvent molecules into the plant material. This also shortens the diffusion path of solutes, accelerating the dissolution of active components [22,23]. However, when particles become excessively fine, further improvements in extraction efficiency are limited. Thus, moderate grinding to approximately 40 mesh is considered optimal for total alkaloid extraction from *Zanthoxylum bungeanum*.

### 2.2. Orthogonal Design: Results and Range/ANOVA Analysis

Table 1 presents the L9 (3^4^) orthogonal design used to optimize four factors: (A) Particle Size (mesh), (B) Ethanol Concentration, (C) Solid–Liquid Ratio, and (D) pH.

The range analysis results of the orthogonal experiment indicated that the order of factor importance affecting the yield was: A (Particle Size) > D (pH) > B (Ethanol Concentration) > C (Solid–Liquid Ratio). Among these, Factor A had the largest range and was identified as the key factor influencing the process. Although the Analysis of Variance (ANOVA, Table 2) showed that none of the factors reached statistical significance (primarily due to the lack of replication in the saturated design, which limits the estimation of error), the model demonstrated an excellent fit (R^2^ = 0.919), indicating that it explains a substantial proportion of the variability in the response variable. Consequently, the process parameters were optimized based on the sequence-of-importance and the optimal levels identified by the range analysis.

Table 3 summarizes the validation results under the confirmed optimal conditions (A_3_B_2_C_2_D_1_). The scale-up extraction (500 g) demonstrated an average alkaloid yield of 0.465% ± 0.006% with excellent reproducibility (RSD < 1.5%). Compared with the best result obtained from the preliminary orthogonal tests (A_3_B_3_C_2_D_1_), the yield was significantly improved by 7.9%, confirming the superior efficiency and stability of the optimized large-scale process.

Following the optimization of process parameters, the optimized conditions struck a reasonable balance between extraction efficiency and resource consumption, representing a significant improvement over the preoptimization yield. The optimized process not only demonstrated high reproducibility but also showed the versatility of the acidified ethanol–water system for extracting plant alkaloids, thereby underscoring its practical significance.

### 2.3. Purification of Zanthoxylum bungeanum Alkaloids by Macroporous Adsorption Resins

#### 2.3.1. Evaluation of Resin Adsorption Capacity and Selectivity

Following the determination of the optimal extraction conditions, the adsorption performance of the *Zanthoxylum bungeanum* alkaloid crude extract was evaluated using static adsorption on resins. The adsorption capacities of three different resins are summarized in Table 4, with NKA-9 showing the highest capacity of 14.89 mg/g. NKA-9 is a polar macroporous resin, and its polar nature facilitates the selective adsorption of alkaloids. Similar macroporous resin-based purification strategies have been applied to bioactive compounds from *Zanthoxylum* species [25]. 

#### 2.3.2. Elution Process and Purification Efficiency

The adsorbed alkaloids on each resin were eluted stepwise with 50%, 70%, and 90% ethanol (4 bed volumes [BV] each). The alkaloid content in each fraction was determined (Table 5). The results indicated that all three resins effectively adsorbed the alkaloids. Among them, the NKA-9 resin showed superior performance, yielding the highest alkaloid content (5.97%) in the 70% ethanol eluate fraction.

NKA-9 was identified as a medium-polarity macroporous resin with a specific surface area and pore size similar to those of D101, while differing slightly in surface functional groups, which may enhance its affinity for alkaloids [25]. NKA-9 was therefore selected for the purification of *Zanthoxylum bungeanum* alkaloids.

Although the 70% ethanol eluate fraction yielded a lower dry weight (Table 6), it exhibited the best performance in both the yield and recovery rate of total alkaloids. Furthermore, the resulting extract was lighter in color, indicating superior impurity removal. This high purity likely contributed to its excellent biological activity, making the 70% ethanol eluate fraction the optimal segment for enriching the target amide alkaloids. Consequently, subsequent in vitro antioxidant and antibacterial activity studies were focused on this active fraction.

### 2.4. Antioxidant Activities of Zanthoxylum bungeanum Alkaloids

#### 2.4.1. DPPH· Radical Scavenging

*Zanthoxylum bungeanum* is rich in alkaloids, particularly alkylamides [26]. Therefore, this study investigated the free radical scavenging activity (RSA) of *Zanthoxylum bungeanum* alkaloids. Previous studies have reported that, due to the diverse mechanisms underlying free radical scavenging, the antioxidant activity of these alkaloids cannot be accurately assessed using a single assay; hence, multiple assays are recommended [27].

**Figure 2:**
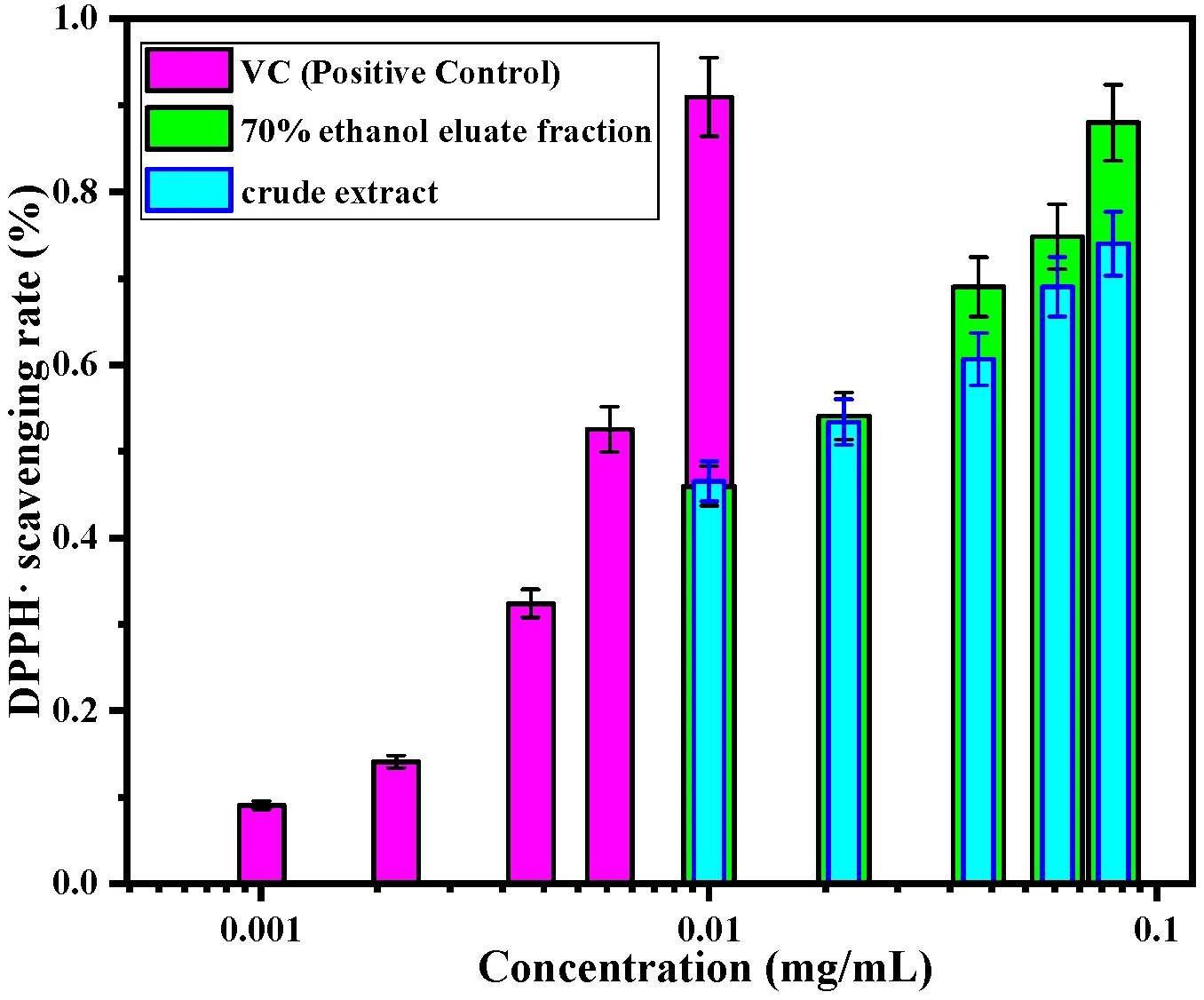
DPPH· radical scavenging activity of *Zanthoxylum bungeanum* alkaloid.

DPPH· is a stable free radical frequently used to assess the antioxidant activity of natural compounds. Its scavenging activity is primarily attributed to its hydrogen-donating ability [28,29]. As shown in Figure 2 and Table 7, the scavenging effects of the *Zanthoxylum bungeanum* alkaloid crude extract, the 70% ethanol eluate fraction, and vitamin C on DPPH· radicals were concentration-dependent, with the scavenging rate increasing approximately linearly with increasing concentration. Based on regression analysis, the IC_50_ of the 70% ethanol eluate fraction was approximately 0.013 mg/mL, slightly higher than that of vitamin C (0.0057 mg/mL), indicating that its scavenging capacity corresponds to 43.84% that of vitamin C at equivalent concentrations. In the DPPH· assay, antioxidants act by donating hydrogen atoms or electrons to neutralize free radicals. Their activity is primarily influenced by molecular structure and electron cloud distribution.

#### 2.4.2. ABTS^+^· Radical Scavenging

**Figure 3:**
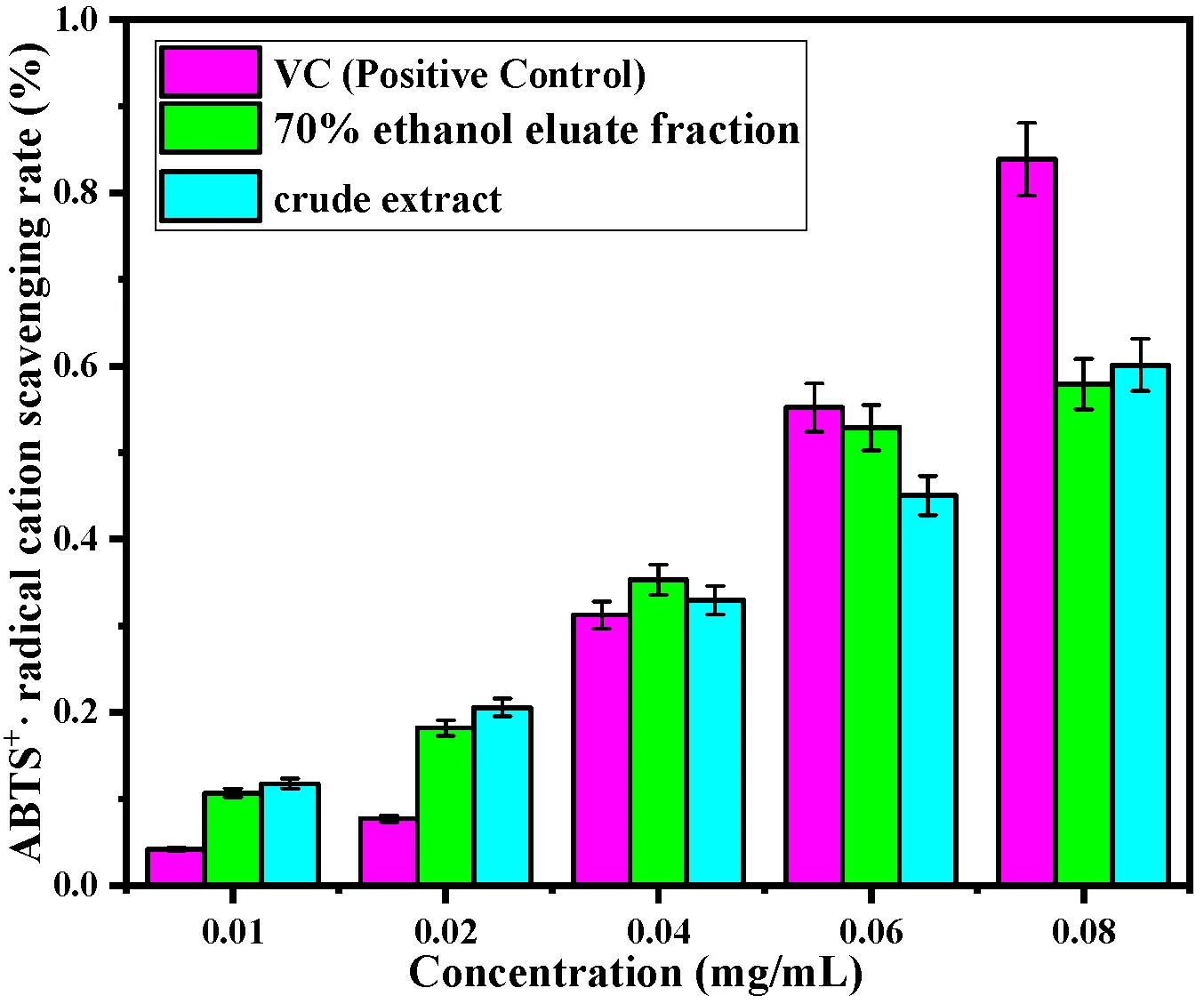
ABTS^+^· radical scavenging activity of *Zanthoxylum bungeanum* alkaloid.

In addition to the DPPH· assay, the ABTS^+^· assay is evaluated the antioxidant activity of natural compounds [30,31]. Figure 3 and Table 8 demonstrate that the *Zanthoxylum bungeanum* alkaloids crude extract, 70% ethanol eluate fraction and vitamin C exhibited concentration-dependent ABTS^+^· scavenging activity. The IC_50_ of the 70% ethanol eluate fraction was approximately 0.063 mg/mL, slightly higher than that of vitamin C (0.054 mg/mL). The ABTS^+^·assay is effective for both hydrophilic and lipophilic antioxidants and is therefore particularly suitable for evaluating the antioxidant activity of moderately polar compounds, such as *Zanthoxylum bungeanum* alkaloids.

#### 2.4.3. Reducing Power Determination

**Figure 4:**
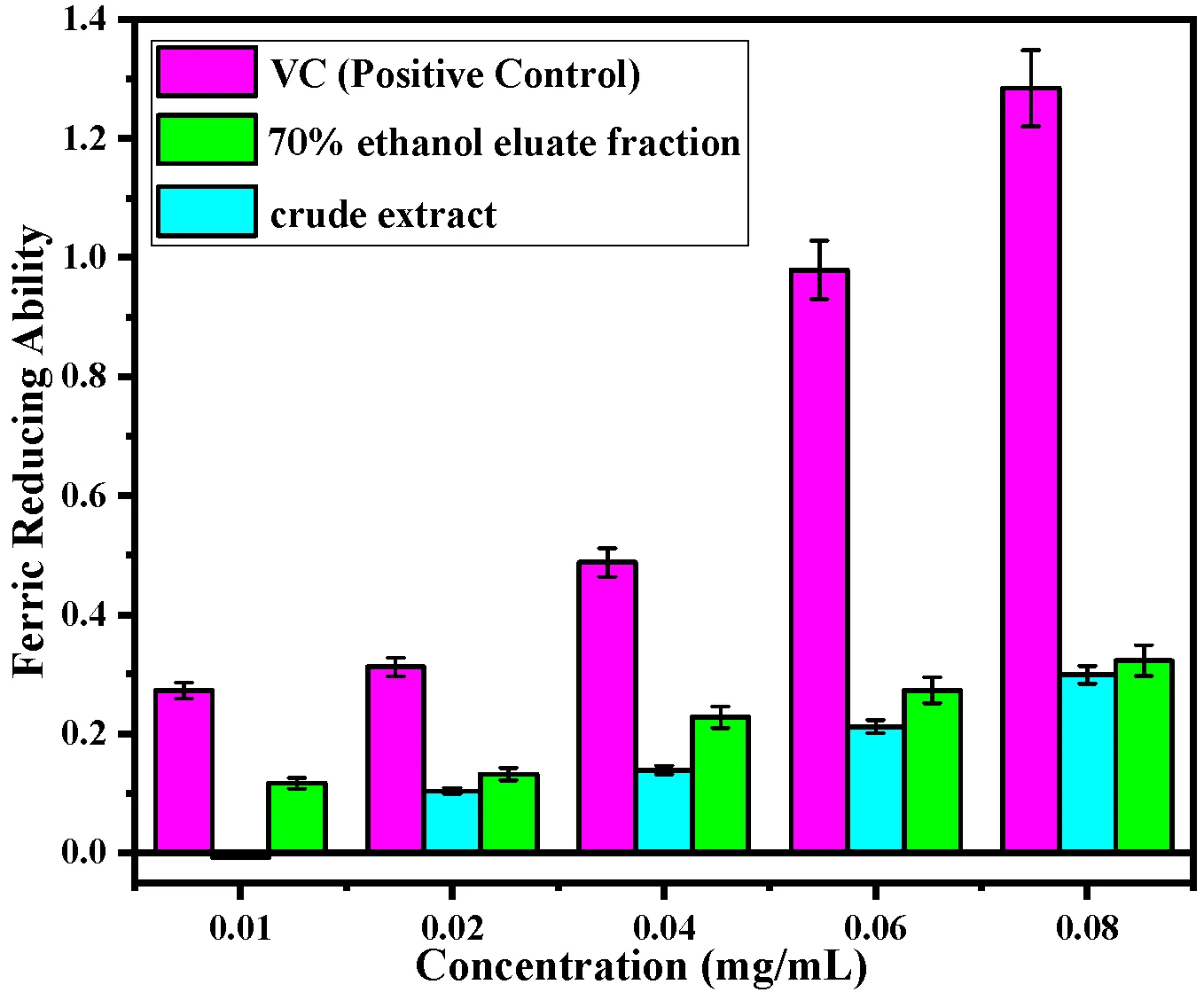
Fe^3+^-reduction activity of *Zanthoxylum bungeanum* alkaloid.

In 2015, Gülçin [32] comprehensively reviewed the reduction in Fe^3+^ to Fe^2+^ by antioxidants. This Fe^3+^ reducing ability is commonly used to assess the reducing capacity of food components or plant extracts. However, the reducing capacity of these bioactive compounds reflects their electron-donating ability, which correlates with their antioxidant activity [33]. As shown in Figure 4 and Table 9, the absorbance at 700 nm increased with increasing sample concentration, indicating enhanced formation of the Prussian blue complex and corresponding increases in reducing power. The EC_50_ of the 70% ethanol eluate fraction was approximately 0.131 mg/mL, slightly higher than that of vitamin C (0.031 mg/mL). In this study, the 70% ethanol eluate fraction exhibited the capacity to reduce Fe^3+^ to Fe^2+^. This reduction correlated closely with the alkaloid content of the samples, suggesting synergistic interactions among the purified components.

### 2.5. Antibacterial Activities of Zanthoxylum bungeanum Alkaloids

#### 2.5.1. Qualitative Analysis: Inhibition Zone Diameters

**Figure 5:**
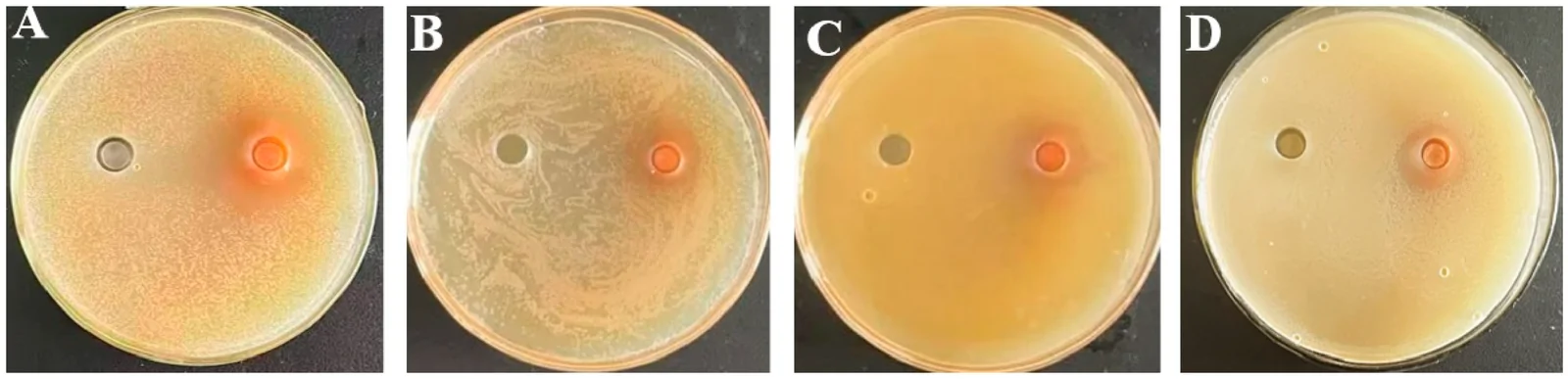
Representative photographs of inhibition zones produced by *Zanthoxylum bungeanum* alkaloid (10 mg/mL) against four bacterial strains using the Oxford cup method. (**A**) *Escherichia coli*; (**B**) *Bacillus subtilis*; (**C**) *Staphylococcus aureus*; (**D**) *Pseudomonas aeruginosa*. Solvent control (DMSO) showed no inhibition zone.

Based on the comparative analysis of antioxidant activity, the 70% ethanol eluate fraction demonstrated higher activity per unit mass than the crude extract, indicating that the purification process effectively concentrated the bioactive components. Therefore, to more efficiently assess the core antibacterial potential of this active fraction, subsequent experiments focused on evaluating the 70% ethanol eluate fraction. The Oxford cup method was employed to determine the diameter of the inhibition zones, and the microbroth dilution method was used to determine the minimum inhibitory concentration (MIC) and minimum bactericidal concentration (MBC). Figure 5 and Table 10 summarize the antibacterial activity of *Zanthoxylum bungeanum* alkaloid (10 mg/mL) against the four tested strains. Clear inhibition zones were evident for all strains, while the solvent control (DMSO) produced no zone, indicating no solvent interference. Table 10 lists the inhibition zone diameters: 20.27 ± 0.25 mm for *E. coli*, 17.27 ± 0.16 mm for *B. subtilis*, 16.67 ± 0.20 mm for *S. aureus*, and 15.93 ± 0.17 mm for *P. aeruginosa*. *E. coli* showed the largest zone and *P. aeruginosa* the smallest, with a difference of approximately 4.34 mm. The zones for *B. subtilis* and *S. aureus* were similar, differing by 0.60 mm. Standard deviations were all below 0.25 mm, indicating good reproducibility. These data show that 10 mg/mL *Zanthoxylum bungeanum* alkaloid inhibits all four strains, with the strongest effect against *E. coli* and the weakest against *P. aeruginosa*, consistent with strain-dependent activity.

#### 2.5.2. Quantitative Analysis I: MIC Determination and Bacterial Growth Curves

**Figure 6:**
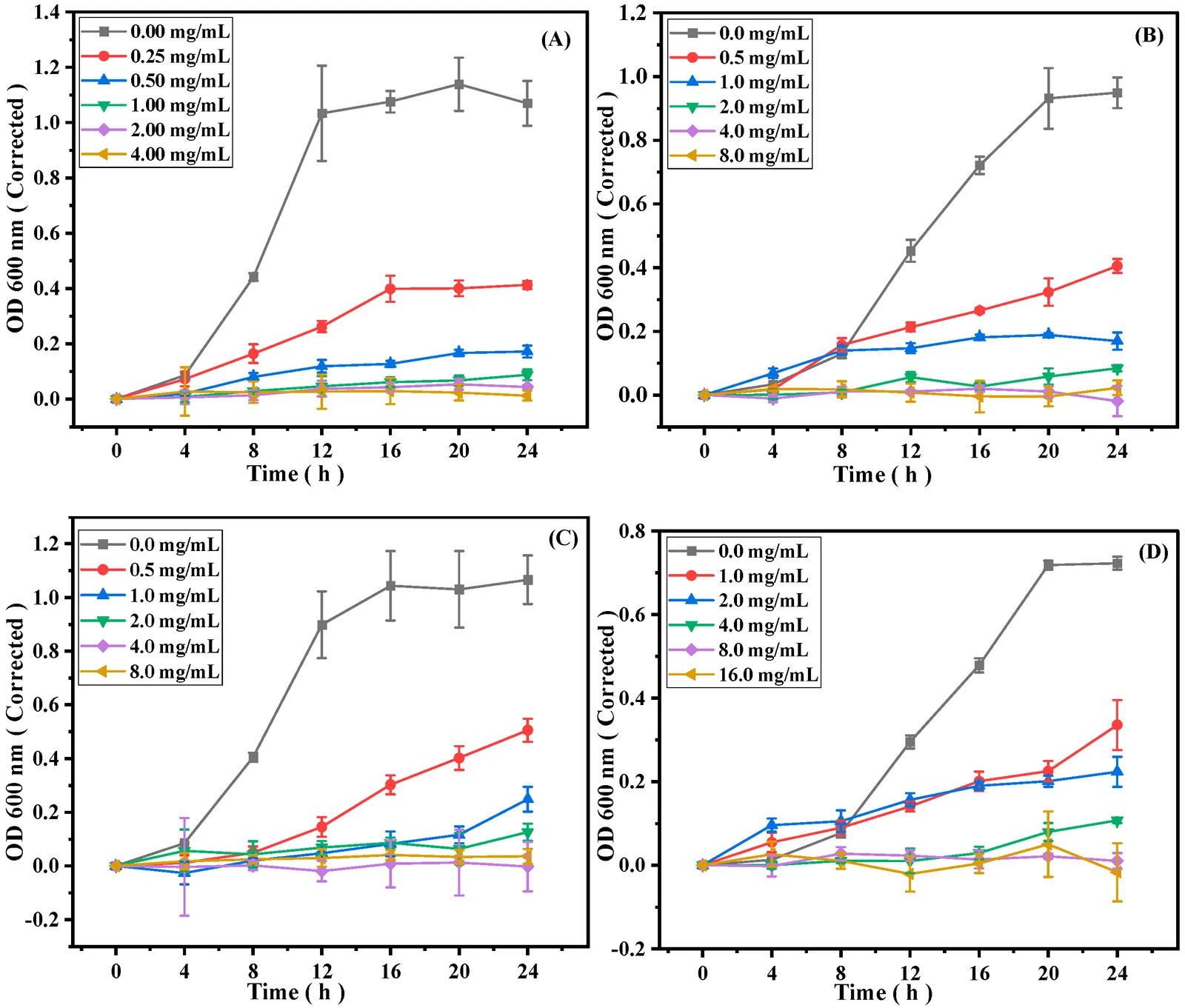
Growth curves of four bacterial strains exposed to varying concentrations of *Zanthoxylum bungeanum* alkaloids. Growth was assessed by OD_600_ measurements every 4 h over 24 h. Data were corrected for background absorbance (OD_600_ at 0 h). Symbols denote mean values (n = 3), and error bars show standard deviation. The minimum inhibitory concentration (MIC) for each strain is indicated. (**A**) *Escherichia coli* (MIC = 2 mg/mL); (**B**) *Bacillus subtilis* (MIC = 4 mg/mL); (**C**) *Staphylococcus aureus* (MIC = 4 mg/mL); (**D**) *Pseudomonas aeruginosa* (MIC = 8 mg/mL). Control (0 mg/mL); other symbols correspond to the concentrations indicated in each panel.

Minimum inhibitory concentrations (MICs) of *Zanthoxylum bungeanum* alkaloid against *E. coli*, *B. subtilis*, *S. aureus*, and *P. aeruginosa* were determined via the flask macrobroth dilution method, and bacterial growth was monitored by measuring optical density at 600 nm (OD_600_) every 4 h over 24 h to generate growth curves (Figure 6). The 70% ethanol eluate fraction inhibited the growth of all four strains in a concentration-dependent manner. For *E. coli* (Figure 6A), partial inhibition was observed at 0.25 and 0.5 mg/mL, pronounced inhibition at 1 mg/mL, and complete suppression at 2 and 4 mg/mL, yielding an MIC of 2 mg/mL. For *B. subtilis* (Figure 6B), partial inhibition was observed at 0.5 and 1 mg/mL, greater inhibition at 2 mg/mL, and complete suppression at 4 and 8 mg/mL, yielding an MIC of 4 mg/mL. For *S. aureus* (Figure 6C), partial inhibition at 0.5 and 1 mg/mL, marked inhibition at 2 mg/mL, and complete suppression at 4 and 8 mg/mL were observed, yielding an MIC of 4 mg/mL. *P. aeruginosa* (Figure 6D) was less susceptible, showing partial inhibition at 1 and 2 mg/mL, enhanced inhibition at 4 mg/mL, and complete suppression at 8 and 16 mg/mL, resulting in an MIC of 8 mg/mL. These results demonstrate that *Zanthoxylum bungeanum* alkaloid inhibits both Gram-positive and Gram-negative bacteria, with the greatest potency against *E. coli* (MIC = 2 mg/mL) and comparatively weaker activity against *P. aeruginosa* (MIC = 8 mg/mL).

#### 2.5.3. Quantitative Analysis II: MBC Determination

The MBC results are shown in Table 11. For the positive control (0 mg/mL), bacterial suspensions were diluted 10^−5^ and plated (20 μL/plate). The mean colony counts for *E. coli*, *B. subtilis*, *S. aureus*, and *P. aeruginosa* were 66.7, 98.7, 115.3, and 105.0 CFU/plate, respectively, corresponding to initial inocula of 3.34 × 10^8^, 4.94 × 10^8^, 5.77 × 10^8^, and 5.25 × 10^8^ CFU/mL. The MBC criterion (≥99.9% killing) was defined as surviving cells ≤ 0.1% of the initial inoculum, which translates to thresholds of 6.68, 9.88, 11.54, and 10.50 CFU/plate at a 10^−3^ dilution (20 μL plating), respectively.

For *E. coli*, the mean surviving colony count at 8 mg/mL was 3.0 CFU/plate, which is below the threshold (6.68) and thus meets the killing criterion; counts at 4 mg/mL and lower exceeded the threshold. Therefore, the MBC for *E. coli* is 8 mg/mL. For *B. subtilis*, the mean surviving colony count at 16 mg/mL was 7.3 CFU/plate, below the threshold (9.88), yielding an MBC of 16 mg/mL. By contrast, the surviving colony count for *S. aureus* at 16 mg/mL was 439.0 CFU/plate, well above the threshold (11.54). For *P. aeruginosa* at 32 mg/mL it was 773.3 CFU/plate, well above the threshold (10.50); no MBC was detected (ND) for either strain within the tested concentration range. The MBC/MIC ratios were 4 for *E. coli* (8/2 = 4) and 4 for *B. subtilis* (16/4 = 4), consistent with bactericidal activity, whereas *S. aureus* and *P. aeruginosa* had no detectable MBCs (MBC/MIC > 4), consistent with bacteriostatic activity. Overall, these results indicate that *Zanthoxylum bungeanum* alkaloid is bactericidal against *E. coli* and *B. subtilis* and bacteriostatic against *S. aureus* and *P. aeruginosa*.

#### 2.5.4. Discussion on Antibacterial Activity

We systematically evaluated the antibacterial and bactericidal activities of *Zanthoxylum bungeanum* alkaloid against four common pathogenic bacteria—*Escherichia coli*, *Bacillus subtilis*, *Staphylococcus aureus*, and *Pseudomonas aeruginosa*—using the Oxford cup method, macrobroth dilution, and MBC determination in accordance with CLSI M26-A guidelines [34]. The alkaloid displayed strain-dependent activity, showing the greatest effect against *E. coli* (inhibition zone diameter 20.27 mm; MIC= 2 mg/mL) and the least effect against *P. aeruginosa* (inhibition zone diameter 15.93 mm; MIC = 8 mg/mL). MBC testing indicated bactericidal activity against *E. coli* and *B. subtilis* (MBC/MIC = 4 for both) and only bacteriostatic activity against *S. aureus* and *P. aeruginosa* within the tested concentration range.

The potent activity of the *Zanthoxylum bungeanum* alkaloids of the 70% ethanol eluate fraction against both Gram-negative and Gram-positive bacteria aligns with prior reports on the plant’s antibacterial properties. Phytochemical analyses show that *Zanthoxylum bungeanum* pericarps contain a diverse suite of alkaloids, including eight structurally distinct compounds (**1**–**8**) [35]. Compounds **4** and **20** were reported to inhibit *S. aureus* [35]. Previous studies on individual constituents have reported significant antibacterial activity, finding that compound **7** was highly active against *S. aureus* and that compound **8** exhibited inhibitory activity against *E. coli* with a MIC of 6.25 μg/mL, comparable to the plant alkaloid berberine [36]. In contrast, the MICs observed here implicate *Zanthoxylum bungeanum* alkaloids in the plant’s broad-spectrum antibacterial effects. The MICs observed here for the total alkaloid extract (2–8 mg/mL) exceeded those reported for isolated compounds, as expected, because the crude extract contains both active and inactive constituents.

The greater activity against *E. coli* (MIC = 2 mg/mL) than against *P. aeruginosa* (MIC = 8 mg/mL) is notable and can be attributed to differences in outer membrane properties between these Gram-negative species. *P. aeruginosa* has a characteristically impermeable outer membrane and constitutively expresses multiple efflux pumps, which underlie its intrinsic resistance to many antimicrobials [37]. The MBC data corroborate this view: *E. coli* was completely killed at 8 mg/mL, whereas *P. aeruginosa* remained viable even at 32 mg/mL. In this study, *P. aeruginosa* showed the lowest susceptibility among the four strains tested to the *Zanthoxylum bungeanum* alkaloid extract. Its inhibition zone was 15.93 mm compared with 20.27 mm for *E. coli*. Its MIC was 8 mg/mL (fourfold higher than that for *E. coli*), and no MBC was observed within the tested range (up to 32 mg/mL), indicating a bacteriostatic effect against this strain. This relative insensitivity likely reflects *P. aeruginosa’s* intrinsic defenses, including its impermeable outer membrane, constitutive multidrug efflux systems (e.g., MexAB-OprM), and capacity for biofilm formation [37,38]. These features render *P. aeruginosa* difficult to eradicate, explaining why the alkaloid extract showed only bacteriostatic, not bactericidal, activity against this strain. Likewise, the differing susceptibilities of *B. subtilis* and *S. aureus* (both Gram-positive) likely reflect variations in cell wall composition or distinct resistance mechanisms [39].

**Figure 7:**
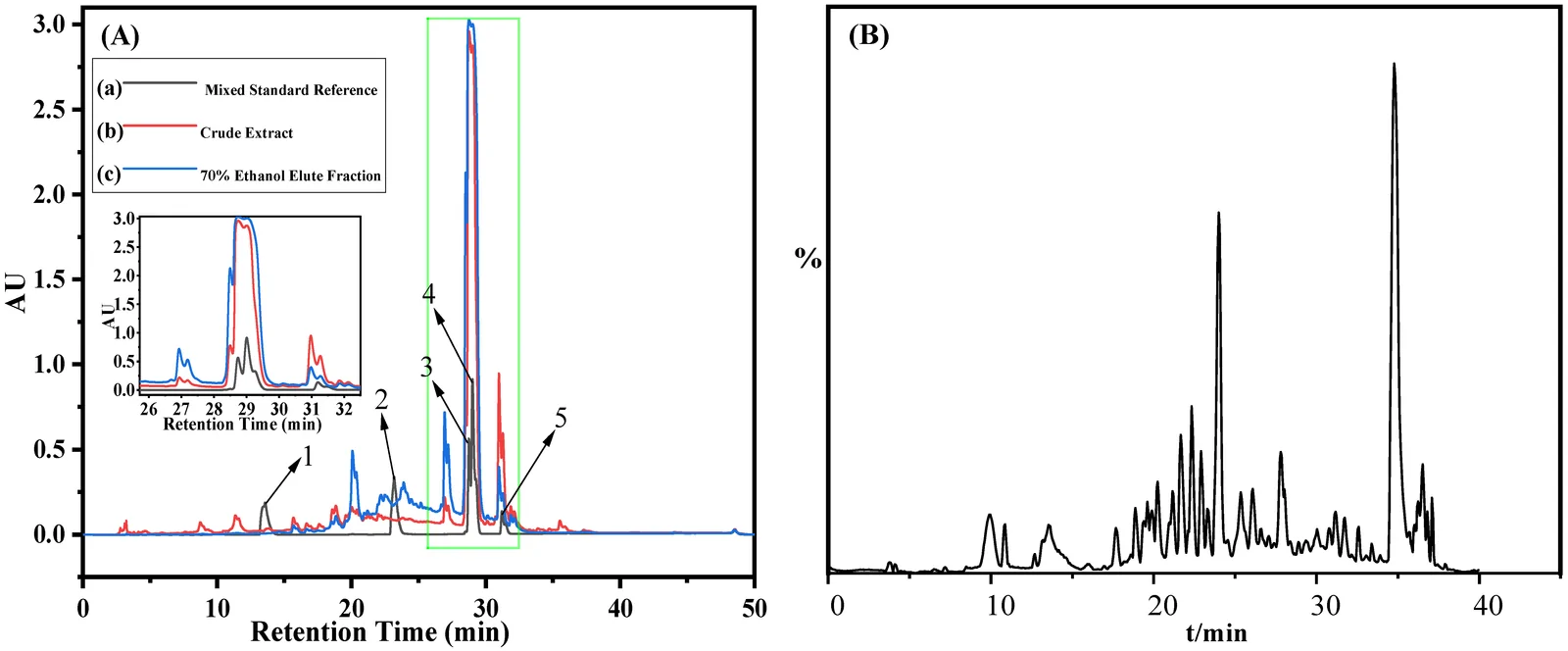
(**A**) UPLC-DAD chromatogram (λ = 280 nm) from the pericarp of *Zanthoxylum bungeanum*. Peaks 1–5 were identified as: 1, sinomenine hydrochloride; 2, chelerythrine hydrochloride; 3, hydroxy-α-sanshool; 4, hydroxy-β-sanshool; 5, rutaecarpine, based on co-injection with authentic standards. The green box indicates the magnified area displayed in the inset. (**B**) Base Peak Intensity (BPI) chromatograms of the 70% ethanol eluate fraction analyzed by UPLC-HRMS in positive ion mode.

**Figure 8:**
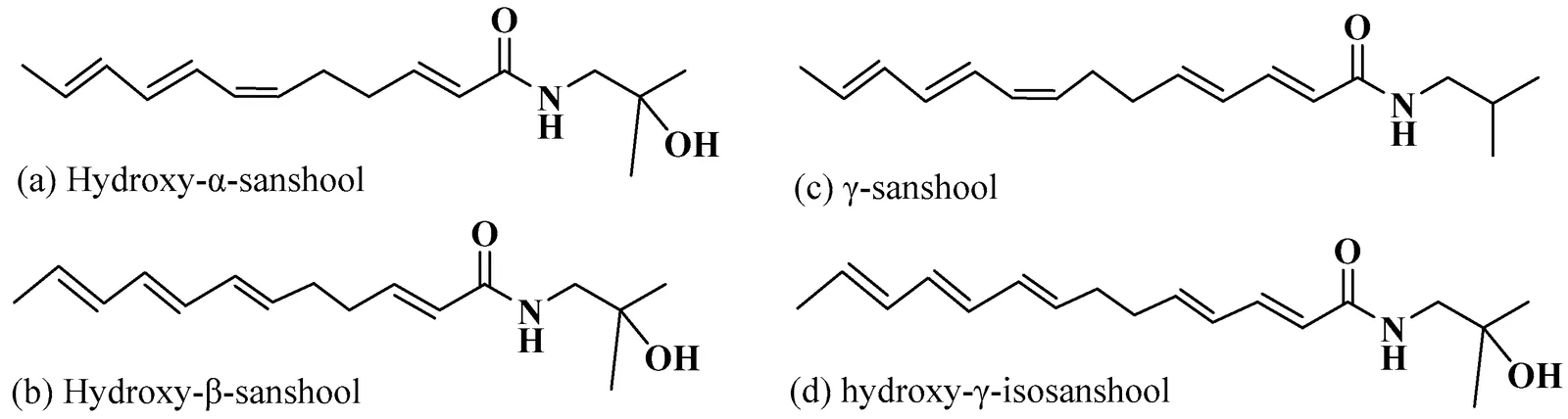
Representative chemical scaffolds of the major alkaloids identified in the 70% ethanol eluate fraction of *Zanthoxylum bungeanum*. (**a**) hydroxy-α-sanshool, (**b**) hydroxy-β-sanshool, (**c**) γ-sanshool (representative alkylamide), (**d**) hydroxy-γ-isosanshool.

The observed strain selectivity can be rationalized by the representative alkaloid scaffolds identified in this study using UPLC-DAD and UPLC-HRMS (Figure 7) and further elucidated structurally in Figure 8. The dominant alkenylamides (e.g., hydroxy-α-sanshool, γ-sanshool) are known to disrupt bacterial membranes via their hydrophobic alkyl chains and polar amide heads [36]. This membrane-active mechanism explains the stronger bactericidal effect against *E. coli* (more permeable outer membrane) compared with the bacteriostatic effect against *P. aeruginosa* (impermeable outer membrane + constitutive efflux) [37,38]. Furthermore, the presence of co-occurring benzophenanthridine-type alkaloids (e.g., liriodenine, compound **8**) reported in *Zanthoxylum bungeanum* pericarps [36] may contribute additional oxidative stress, enhancing overall antibacterial potency.

The MBC/MIC ratio is commonly used to classify antimicrobials as bactericidal (MBC/MIC ≤ 4) or bacteriostatic (MBC/MIC > 4) [34]. By this criterion, the *Zanthoxylum bungeanum* alkaloid of the 70% ethanol eluate fraction was bactericidal against *E. coli* and *B. subtilis* (MBC/MIC = 4 for both) but only bacteriostatic activity against *S. aureus* and *P. aeruginosa* (no MBC detected up to the highest tested concentrations). This differential activity could have clinical implications: the alkaloid may be more appropriate for infections caused by *E. coli* and *B. subtilis* when eradication is required, whereas for *S. aureus* and *P. aeruginosa* it may be more effective as a growth inhibitor than a killing agent.

The strain-dependent activity observed in this study is consistent with the established view that Gram-negative bacteria are often less susceptible to some plant-derived antimicrobials due to their outer membrane barrier [38]. The greater susceptibility of *E. coli* compared with *P. aeruginosa* indicates substantial variation within the Gram-negative group, likely reflecting differences in outer membrane composition, efflux pump expression, and target-site accessibility [37,38].

Several limitations of this study warrant acknowledgment. First, the alkaloid of the 70% ethanol eluate fraction tested was a total extract rather than purified individual compounds. Future work should identify the specific alkaloid(s) responsible for the observed activity and characterize their structure–activity relationships. Second, these experiments were performed in vitro; in vivo studies in appropriate animal infection models are needed to determine pharmacokinetics, toxicity, and therapeutic efficacy. Third, the mechanisms that underlie the different susceptibilities of the four tested strains—specifically, the bactericidal activity against *E. coli* and *B. subtilis* versus the bacteriostatic effect against *S. aureus* and *P. aeruginosa*—require further investigation. Fourth, this study did not include a standard antibiotic positive control (e.g., gentamicin). The MIC and MBC values obtained according to CLSI guidelines are internationally accepted quantitative endpoints and can be interpreted without a positive control. In addition, the DMSO solvent control showed no inhibition zones for any tested strain, indicating the absence of solvent-related effects. Nevertheless, future studies should incorporate a positive control to validate the extract’s relative potency.

In conclusion, the *Zanthoxylum bungeanum* alkaloid of the 70% ethanol eluate fraction displays pronounced, strain-dependent antibacterial and bactericidal effects against four common pathogenic bacteria. Activity was strongest against *E. coli*, and the alkaloid showed bactericidal effects against both *E. coli* and *B. subtilis*. These results support the potential use of the *Zanthoxylum bungeanum* alkaloid as a natural antibacterial agent, especially for infections caused by *E. coli* and *B. subtilis*. Further work is needed to identify the active constituents, clarify their mechanisms of action, and assess their safety and efficacy in vivo.

### 2.6. Identification of Chemical Constituents in the Active Fraction

Although the 70% ethanol eluate fraction did not yield the highest dry weight, it demonstrated the highest alkaloid content and recovery rate, along with superior purity. Combined with the preliminary indication that it is enriched with characteristic bioactive alkaloids (Table 12), this fraction was unequivocally selected for all subsequent in vitro antioxidant and antibacterial studies. To elucidate the chemical basis of the 70% ethanol eluate fraction and systematically evaluate the purification efficiency of NKA-9 resin, this study employed an integrated analytical approach using ultra-performance liquid chromatography coupled with ultraviolet detection (UPLC-UV) and high-resolution mass spectrometry (UPLC-IM-QTOF/MS). Detailed chromatographic and mass spectrometric conditions are provided in the Supplementary Information. First, UPLC-UV analysis was performed on five mixed standard references (a), the crude extract (b), and the 70% ethanol eluate fraction (c) (Figure 7A). The results clearly demonstrated that, compared with the highly complex crude extract (b), the 70% ethanol eluate fraction (c) showed a significant reduction in the number of chromatographic peaks, while the signals of target peaks (e.g., tR = 28.68 min and tR = 28.98 min) were substantially enhanced, indicating that the purification process effectively removed impurities and enriched the target constituents. More importantly, the retention times of the major peaks in the 70% ethanol eluate fraction (c) completely matched those of the corresponding reference standards (a), providing preliminary evidence for the identification of the key components.

To confirm the chemical structures, we further obtained the base peak intensity (BPI) chromatograms of the 70% ethanol eluate fraction and the crude extract (Figure S1) in positive ion mode via high-resolution mass spectrometry (Figure 7B), and systematically identified the amide alkaloids in both the crude extract and the 70% ethanol eluate fraction. The comparison clearly showed that the BPI chromatogram of the crude extract (Figure S1) exhibited a complex background with high baseline noise, whereas the ion current signals of the target compounds in the 70% ethanol eluate fraction were significantly enhanced, and its spectrum appeared much clearer, once again verifying the purification effectiveness at the mass spectrometry level. By comparing the accurate molecular weights and MS/MS fragment ion information, the major components were successfully identified (with identification data for the crude extract provided in Supplementary Table S3).

In this study, 11 putative major alkaloids were successfully identified from the 70% ethanol eluate fraction (Table 12), including key components such as hydroxy-α-sanshool and hydroxy-β-sanshool. These compounds have been extensively documented to exhibit significant antibacterial and antioxidant activities. It is noteworthy that, as shown in Supplementary Table S3, various amide alkaloids present in the crude extract were detected in the 70% ethanol eluate fraction, with their mass spectrometric response intensities significantly higher than those in the crude extract (Table S3). This confirms, at the chemical level, that the purification process successfully achieved the targeted enrichment of targeted bioactive constituents.

In summary, through multi-faceted lines of evidence involving standard reference comparison, chromatogram visualization, and high-resolution mass spectrometry (HR-MS) confirmation, this study not only comprehensively elucidated the core chemical constituents of the 70% ethanol eluate fraction, but also comprehensively revealed, at the compositional level, the excellent purification and enrichment capacity of NKA-9 resin. This provides a robust foundation for understanding its potent in vitro bioactivity. The structures of the dominant components are depicted in Figure 8.

## 3. Materials and Methods

### 3.1. Materials and Reagents

This study used red *Zanthoxylum bungeanum* pericarp (carefully sourced from Longnan Longxiangyuan Agricultural Products Development Co., Ltd., Longnan, China) as the main raw material.

Reference compounds: Sinomenine hydrochloride (HPLC ≥ 98%, Lot No.: 20260313) was purchased from Xi’an Tianbao Biological Technology Co., Ltd., Xi’an, China; Chelerythrine hydrochloride (HPLC ≥ 98%, Lot No.: DSTDY039002), hydroxy-α-sanshool (HPLC ≥ 98%, Lot No.: DST251213-175), and hydroxy-β-sanshool (HPLC ≥ 98%, Lot No.: DST240202-176) were obtained from Lemeitian Pharmaceutical Biotechnology, Chengdu, China;; Evodiamine (HPLC ≥ 98%, Lot No.: HL-250320) was supplied by Xi’an Huilin Biological Technology Co., Ltd, Xi’an, China.

Reagents: Bromocresol green indicator (pH range: 3.8–5.4) was purchased from Tianjin Zhonglian Chemical Reagent Co., Ltd., Tianjin, China; Acetic acid (Analytical Reagent, AR) was obtained from Fuchun (Tianjin) Chemical Reagent Co., Ltd., Tianjin, China; Sodium acetate anhydrous (CH_3_COONa, AR) was purchased from Xilong Scientific Co., Ltd., Guangzhou, China; Chloroform (AR) was obtained from Chengdu Kelong Chemical Co., Ltd., Chengdu, China; Anhydrous ethanol (Purity ≥ 99.0%) was purchased from Anhui Tiandi High Purity Solvent Co., Ltd., Hefei, China; Acetonitrile (HPLC grade) was purchased from Shanghai Titan Technology Co., Ltd., Shanghai, China.

Antioxidant assay reagents: 1,1-Diphenyl-2-picrylhydrazyl (DPPH, Purity > 98%) was purchased from Shanghai Macklin Biochemical Co., Ltd., Shanghai, China; Ascorbic acid (Purity > 99.0%) was obtained from Shanghai Aladdin Biochemical Technology Co., Ltd., Shanghai, China; 2,2′-Azino-bis(3-ethylbenzothiazoline-6-sulfonic acid) (ABTS, Purity > 98%) was purchased from Tianjin Zhonglian Chemical Reagent Co., Ltd., Tianjin, China; Potassium persulfate (K_2_S_2_O_8_, AR) was obtained from Xi’an Chemical Reagent Factory, Xi’an, China.

Ferric reducing power assay reagents: Potassium ferricyanide (K_3_[Fe(CN)_6_], 1% *w*/*v* solution) and Ferric chloride (FeCl_3_, 0.1% *w*/*v* solution) were purchased from China National Pharmaceutical Group Chemical Reagent Co., Ltd., Beijing, China; PBS buffer (0.2 mol/L, pH 6.6) was purchased from Hefei Bomei Biological Technology Co., Ltd., Hefei, China; Trichloroacetic acid solution (CCl_3_COOH, 10%) was obtained from Tianjin Jinboli Experimental Equipment Sales Co., Ltd., Tianjin, China.

### 3.2. Instruments and Equipment

The instruments used included a 754 UV-Vis spectrophotometer (Shanghai Jinghua Technology Instrument Co., Ltd., Shanghai, China, data acquisition for the UV-Vis spectrophotometer was performed using the instrument-bundled software.), a Mettler TLE204 electronic analytical balance (precision: 0.1 mg, Mettler-Toledo Instruments (Shanghai) Co., Ltd., Shanghai, China), a THZ-82AHS air-bath constant temperature oscillator (Changzhou Guoyu Instrument Manufacturing Co., Ltd., Changzhou, China), a Harris RH-20 grinder, a KQ-500DE CNC ultrasonic cleaner (Kunshan Ultrasonic Instrument Co., Ltd., Kunshan, China), and a Bio-Rad gel documentation system (Bio-Rad Laboratories, Inc., Hercules, CA, USA).

### 3.3. Experimental Methods

#### 3.3.1. Establishment of the Standard Curve

The total alkaloid content (TAC) of the extracts was determined by a modified acid–dye colorimetric method based on the procedure reported by Tian et al. [40]. In the experiment, 0.0080 g of chelerythrine reference standard was accurately weighed into a 25 mL volumetric flask. An appropriate amount of ethanol was then added to dissolve the standard under ultrasonic treatment, and the volume was made up to the mark to obtain a 0.32 mg/mL stock solution.

Volumes of 1, 2, 3, 4, and 5 mL of the prepared stock solution were accurately pipetted into separate 20 mL stoppered test tubes, which were labeled 1–5. Then, 2 mL of bromocresol green phosphate buffer (pH ≈ 3.6) and 10 mL of chloroform were added; the mixture was thoroughly shaken, and the volume was adjusted with purified water. The tubes were sealed and shaken vigorously for 1–2 min to facilitate the extraction and formation of the alkaloid–acid dye complex. The mixtures were transferred into separatory funnels and allowed to stand for 1 h until phase separation occurred. The chloroform layer was collected and dried over anhydrous sodium sulfate. The lower chloroform phase was scanned spectrophotometrically over 400–600 nm to obtain its absorption spectrum. The maximum absorption wavelength after color development was determined to be λmax = 430 nm. Before this operation, the chloroform phase devoid of alkaloids but with the same color development treatment was used as a blank for baseline adjustment.

**Figure 9:**
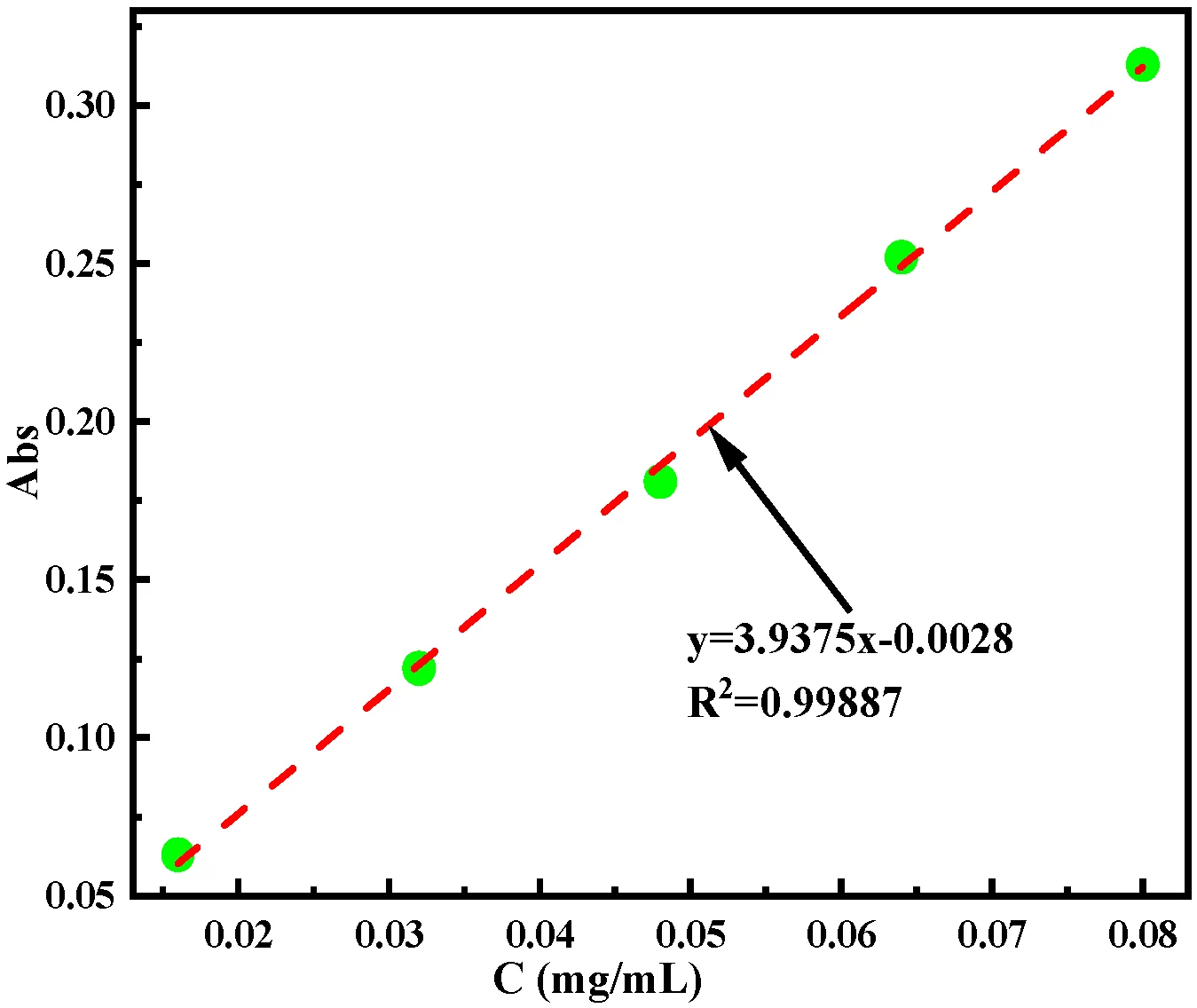
Standard Curve.

The absorbance values of solutions 1–5 were recorded at λ_max_, and standard solutions with varying concentrations were prepared using chelerythrine as the reference compound. A calibration curve of absorbance (y) versus alkaloid concentration (x) was constructed, yielding the regression equation y = 3.9375x − 0.0028 (R^2^ = 0.9989) within the concentration range of 0.016–0.080 mg/mL (Figure 9).

The determination of the total alkaloid yield was conducted based on the standard curve method mentioned above. Two milliliters of the extraction solution (after being diluted to a fixed volume of 300 mL) was accurately transferred into a 20 mL stoppered graduated test tube for alkaloid content analysis. Two milliliters of bromocresol green phosphate-buffered solution and 10 mL of chloroform were added, and the mixture was diluted to 20 mL with ethanol. The test tube was sealed and shaken vigorously for 1–2 min. After standing, the lower chloroform phase was collected and its absorbance was measured at the maximum absorption wavelength. Before measurement, an extraction solution that had undergone the same color development treatment but contained no alkaloids was used as a blank for correction.

According to the aforementioned method, the yield of alkaloids from *Zanthoxylum bungeanum* was determined under different extraction conditions. The yield (%) was calculated as follows: Y (%) = (Wa/Wp) × 100%, where Y is the alkaloid yield (%), Wa is the total alkaloid content (g), and Wp is the mass of the *Zanthoxylum bungeanum* powders (g).

#### 3.3.2. Optimization of the Extraction Process

The extraction of *Zanthoxylum bungeanum* alkaloids was carried out according to previously reported procedures [2,41], with minor modifications. To investigate the effects of different factors on the extraction efficiency of alkaloids, two sets of experiments were conducted: (a) a single-factor experiment and (b) an orthogonal experiment.

(a) Single-factor experiment:

Effect of solvent concentration: 15.0 g of *Zanthoxylum bungeanum* powders, previously ground and passed through a 20-mesh sieve, was placed in a 500 mL round-bottom flask. The solid–liquid ratio was maintained at 1:15 (225 mL of solvent), and the extraction was performed three times, each for 2 h at 60 °C under reflux. Ethanol concentrations of 50%, 65%, 80%, and 95% (*v*/*v*) were employed to assess the influence of solvent strength on alkaloid extraction efficiency. All solvents were adjusted to pH 3 prior to use. After extraction, the three filtrates were combined, concentrated under reduced pressure, and diluted to 300 mL with ethanol.

Effect of solid–liquid ratio: 15.0 g of *Zanthoxylum bungeanum* powders was placed in a dry flask. Reflux extraction was carried out at different solid-to-liquid ratios (1:10, 1:15, 1:20, and 1:25). The ethanol concentration was fixed at 65% (adjusted to pH 3), and the extraction was performed three times, each for 2 h at 60 °C under reflux. Subsequent operations were performed as described above.

Effect of extract pH: Similarly, 15.0 g of *Zanthoxylum bungeanum* powders was placed in a dry flask. The ethanol concentration was fixed at 65%, and the solid–liquid ratio was set at 1:15. The extraction was performed three times, each for 2 h at 60 °C under reflux. The initial pH of the extraction solvent was adjusted to 2, 3, 4, and 5 with hydrochloric acid before extraction. The alkaloid yields under different pH conditions were determined, and all subsequent procedures were carried out as described previously.

Effect of particle size: *Zanthoxylum bungeanum* powders with particle sizes of 10, 20, 30, and 40 mesh was weighed (15.0 g each) to compare the influence of the degree of grinding on alkaloid yield. The extraction conditions were fixed at 65% ethanol (pH 3), a solid–liquid ratio of 1:15, and extractions were performed three times for 2 h at 60 °C under reflux. All other operations were performed as described above.

(b) Orthogonal experiment:

Based on the results of the single-factor experiments, three of these factors were selected, excluding the least influential variable, for an L9 (3^4^) orthogonal design (Table 13). In the orthogonal experiment, the mass of *Zanthoxylum bungeanum* powders was fixed at 15 g, and the experimental procedure remained identical to the previous method. A blank experiment (without adding *Zanthoxylum bungeanum* powders) was also conducted under the same extraction conditions to verify the influence of solvent residues on the determination results.

The results of the orthogonal experiment were analyzed using analysis of variance (ANOVA) and range analysis to determine the relative importance of the factors and identify the optimal parameter combination. Under the optimal conditions, the extraction was repeated three times, with each extraction lasting 2 h at 60 °C under reflux, and the total alkaloid yield was calculated to verify the optimal extraction efficiency.

#### 3.3.3. Purification of Crude Extract Using Macroporous Resin

Resin selection and pretreatment: Three types of macroporous adsorption resins (D101, AB-8, and NKA-9) were soaked in 95% ethanol to ensure complete swelling. The resins were then rinsed with 95% ethanol to remove impurities (including porogens) until the eluate became clear, after which they were washed with deionized water.

Resin regeneration: The resins were soaked in 5% HCl and 5% NaOH solutions, respectively, for 2–3 h, with occasional shaking to activate the functional groups. Finally, the resins were repeatedly rinsed with deionized water until a neutral pH was reached and no odor was detectable. The wet resins were stored in ethanol for subsequent experiments.

Static adsorption and desorption experiments: An accurately weighed 1.0 g sample of each resin was placed in a 100 mL conical flask. Then, 15 mL of crude extract (prepared by dissolving 0.75 g of dried crude extract in 500 mL, i.e., 1.5 mg/mL) was added, and the mixture was shaken in a thermostatic air bath shaker at 25 °C and 100 rpm for 3 h, followed by static incubation for 6 h. After removing surface moisture from the filtered resin, desorption was performed as follows: The resin was transferred to a 100 mL conical flask, mixed with 30 mL of 95% ethanol, and shaken in a thermostatic air bath shaker at 25 °C and 100 rpm for 3 h, followed by incubation for 6 h. Subsequently, the concentrations of alkaloids in the initial loading solution (C_0_), the post-adsorption filtrate (C_1_), and the desorbate (C_2_) were determined spectrophotometrically after appropriate dilution. Finally, the static adsorption capacity, adsorption rate, and desorption rate for each resin were calculated.

Adsorption and elution: The three pretreated resins were packed into a glass chromatographic column (4.0 cm in diameter × 60 cm in height). The resin bed height was approximately 40 cm, and the wet-packing method was employed to remove air bubbles and prevent the formation of dry cracks. Then, a 15 mg/mL crude extract solution was prepared (by dissolving 7.5 g of dried crude extract in 500 mL, i.e., 15 mg/mL). Then, 500 mL of this solution was loaded onto each of the D101, AB-8, and NKA-9 resin columns for adsorption. The flow rate was maintained at 4 bed volumes per hour (BV/h). After the entire sample solution had passed through, sugars and other impurities were removed with 4 BV of deionized water, followed by stepwise elution with 4 BV each of 50%, 70%, and 90% ethanol. The eluates from each section were collected, concentrated under reduced pressure to remove ethanol, and dried to obtain alkaloid-enriched products for each elution condition. The alkaloid content of each fraction was quantified, and the percentage of the total alkaloid content relative to the initial crude extract was calculated for each fraction.

Analysis of resin purification effect: The alkaloid contents of the 50%, 70%, and 90% ethanol eluates were quantified using the standard curve method, and the recovery rate was calculated as the proportion of the total alkaloid content relative to that in the initial crude extract. A comparative analysis of adsorption capacity and elution efficiency among the three resins was conducted to identify the most suitable resin for alkaloid separation and purification. The resin demonstrating the highest separation efficiency and recovery was subsequently employed for the purification and preparation of total alkaloid samples.

### 3.4. Antioxidant Activity Assay

#### 3.4.1. DPPH· Radical-Scavenging Activity

The experimental procedure was adapted from previously reported methods [27,42,43], with slight modifications. A DPPH· solution (25 mg/L) was prepared by accurately weighing 2.5 mg of DPPH· powder, dissolving it in an appropriate amount of anhydrous ethanol, and bringing the volume up to the mark with ethanol, followed by thorough mixing. DPPH· is photosensitive and should be used immediately or stored in the dark at 4 °C (generally within 24 h).

For the sample preparation, 4.0 mg of either the crude extract or the 70% ethanol eluate fraction was accurately weighed, transferred to a 10 mL volumetric flask, and brought up to the mark with ethanol to obtain a 0.4 mg/mL stock solution. A series of working solutions (0.01, 0.02, 0.04, 0.06, 0.08 mg/mL) was then prepared by diluting the stock solution in 10 mL volumetric flasks. Because vitamin C (VC, ascorbic acid), as a strong reducing agent, exhibits significantly higher radical-scavenging activity than the test samples, a lower concentration series (0.001, 0.002, 0.004, 0.006, 0.01 mg/mL) of its aqueous solution was used to ensure an accurate dose–response curve and reliable IC_50_ calculation.

For the DPPH assay, a clean test tube (or cuvette) was used as the control: 3.9 mL of DPPH· reagent (25 mg/L) was mixed with 0.1 mL of anhydrous ethanol. The mixture was vortexed and kept in the dark at 30 °C for 40 min. Its absorbance at 517 nm (A_control_) was measured using anhydrous ethanol as the blank.

For the sample groups, 15 tubes were labeled separately for the crude extract, the 70% ethanol eluate fraction, and VC, each at its respective concentration. To each sample tube, 1.0 mL of the corresponding sample solution was added, followed by 3.0 mL of DPPH· reagent. After vortex mixing, the tubes were incubated in the dark at 30 °C for 40 min, and the absorbance was measured at 517 nm (A_sample_).

In parallel, another set of 15 tubes (corresponding to the same samples and concentrations) was prepared for background correction: 0.1 mL of each sample solution was mixed with 3.9 mL of anhydrous ethanol. These mixtures were vortexed and their absorbance was measured immediately at 517 nm without incubation (A_blank_).

All measurements were performed in triplicate. The radical-scavenging activity (R, %) was calculated according to the following formula:

R (%) = [1 − (A_sample_ − A_blank_)/A_control_] × 100%.

#### 3.4.2. ABTS^+^·Radical-Scavenging Activity

The experimental procedure was adapted from previously reported methods [27,44,45] with slight modifications. ABTS^+^· was generated by oxidizing ABTS with potassium persulfate. Specifically, 0.384 g of ABTS solid was dissolved in distilled water, transferred to a 100 mL volumetric flask, and diluted to the mark to obtain a 7.00 mmol/L ABTS solution. Separately, 0.0662 g of potassium persulfate was dissolved in distilled water, transferred to a 100 mL volumetric flask, and diluted to the mark to yield a 2.45 mmol/L aqueous solution. Equal volumes of the 7.00 mmol/L ABTS solution and the 2.45 mmol/L potassium persulfate solution were then mixed thoroughly. The mixture was allowed to stand in the dark at room temperature for 16 h to allow complete oxidation of ABTS to ABTS^+^·. For the working solution, approximately 7.14 mL of this ABTS^+^· stock solution was diluted with distilled water in a 250 mL volumetric flask to the mark; the absorbance of the resulting working solution was adjusted to 0.70 ± 0.02 at 734 nm using a UV-Vis spectrophotometer.

For the assay, vitamin C (VC, ascorbic acid) was used as the positive control. Due to its high water solubility, VC was dissolved and diluted with distilled water to prepare aqueous solutions at the same mass concentrations (0.01, 0.02, 0.04, 0.06, 0.08 mg/mL) as those of the crude extract and the 70% ethanol eluate fraction (which were prepared using ethanol as the solvent).

For the ABTS^+^· assay, fifteen test tubes were used. Exactly 3.6 mL of the ABTS^+^· working solution (adjusted to an absorbance of 0.70 ± 0.02) was pipetted into each tube. Then, 0.4 mL of the sample solution at the corresponding concentration—either VC, crude extract, or 70% ethanol eluate fraction—was added. The mixture was vortexed (or shaken) thoroughly and incubated in the dark at room temperature for 20 min. The absorbance of each tube was measured at 734 nm using a UV-Vis spectrophotometer, with distilled water as the blank.

The blank tube contained 3.6 mL of ABTS^+^· solution and 0.4 mL of pure water, and its absorbance was recorded as (A_blank_). The solvent-blank tube contained 3.6 mL of ABTS^+^· solution and 0.4 mL of anhydrous ethanol, and its absorbance was recorded as (A_solvent-blank_). The absorbance of the sample tubes was recorded as (A_sample_), and that of the VC (positive control) tubes as (A_control_). All measurements were performed in triplicate.

The radical-scavenging activity (percentage inhibition) was calculated according to the following formula:

Scavenging Rate (%) = [1 − (A_sample_ − A_solvent-blank_)/(A_control_− A_solvent-blank_)] × 100%.

#### 3.4.3. Fe^3+^ Reducing Power Determination

The Fe^3+^ reducing power was determined according to the methods of Gunathilake [46] and Amarowicz et al. [47], with minor modifications. All reagents other than the self-prepared crude extract and 70% ethanol eluate fraction were commercially purchased as detailed in Section 3.1 (Materials and Reagents). The crude extract, 70% ethanol eluate fraction, and vitamin C (VC, ascorbic acid) were accurately weighed, transferred to volumetric flasks, dissolved, and diluted to the mark with distilled water to prepare their respective stock solutions. A series of working solutions with concentrations of 0.01, 0.02, 0.04, 0.06, and 0.08 mg/mL was then obtained via serial dilution.

For the total reducing power assay, fifteen test tubes were divided into three groups, corresponding to VC, crude extract, and 70% ethanol eluate fraction, respectively. Each group received 0.4 mL of sample solution at one of the following concentrations: 0.01, 0.02, 0.04, 0.06, or 0.08 mg/mL. To each tube, 0.5 mL of phosphate buffer (0.2 mol/L, pH 6.6) and 0.5 mL of potassium ferricyanide solution (1.0%, *w*/*v*) were added. The mixtures were incubated in a 50 °C air-bath shaker for 20 min. After the reaction, the tubes were rapidly cooled in a cold or ice-water bath. Subsequently, 0.5 mL of trichloroacetic acid solution (10%, *w*/*v*) was added to each cooled tube, and the mixture was allowed to stand for 2 min to terminate the reaction. Following this, 0.5 mL of distilled water and 0.5 mL of ferric chloride solution (0.1%, *w*/*v*) were added. The tubes were kept at room temperature for 5 min to allow the complex to develop color. The absorbance of each tube was measured at 700 nm against a distilled-water blank and recorded as (A_sample_). A control tube was prepared by replacing the 0.4 mL sample with 0.4 mL of distilled water while keeping all other steps identical; its absorbance was measured and recorded as (A_control_). In this assay, a higher absorbance (A_sample_) generally indicates stronger reducing power. All measurements were performed in triplicate. The total reducing power was calculated according to the following formula:

R (%) = (A_sample_/A_control_) × 100%.

### 3.5. Antibacterial Activity

#### 3.5.1. Determination of Inhibition Zone Diameters (Oxford Cup Method)

*Escherichia coli* ATCC 9637, *Bacillus subtilis* ATCC 6633, *Staphylococcus aureus* ATCC 25923, and *Pseudomonas aeruginosa* ATCC 27853 were obtained from Beijing Bio-Technology Co., Ltd. (Beijing, China). Strains stored at −80 °C in glycerol were streaked individually onto Luria–Bertani (LB) agar plates and incubated at 37 °C for 18–20 h to revive. Single colonies were inoculated into Mueller–Hinton (M-H) broth and grown at 37 °C with shaking at 180 rpm until the stationary phase (≈18 h for *S. aureus* and *E. coli*; ≈20 h for *B. subtilis* and *P. aeruginosa*). Cultures were adjusted to a 0.5 McFarland standard (≈1.5 × 10^8^ CFU/mL) using sterile physiological saline and then diluted 200-fold in M-H broth to yield a working suspension of ≈7.5 × 10^5^ CFU/mL.

M-H agar was sterilized at 121 °C for 15 min and then cooled to approximately 50 °C. For the lower layer, 15 mL of M-H agar was poured into each sterile 90 mm Petri dish and allowed to solidify at room temperature for 30 min. A separate aliquot of sterilized M-H agar was equilibrated in a 45 °C water bath for 30 min, mixed with the working bacterial suspension in a ratio of 50 μL suspension per 5 mL agar, immediately poured over the solidified lower layer, and gently swirled to ensure uniform distribution. The plates were left to solidify at room temperature for 20 min to yield double-layer plates containing bacteria. Oxford cups (inner diameter 6 mm, outer diameter 8 mm) were placed vertically on the upper-layer surface using sterile forceps and allowed to adhere via gravity without applying pressure. Three cups were evenly arranged on each plate. For the experimental group, 300 μL of a 10 mg/mL *Zanthoxylum bungeanum* alkaloid solution (70% ethanol eluate fraction) (dissolved in DMSO) was added to each cup; the solvent control group received 300 μL of DMSO. Plates were incubated upright at 37 °C for 18–24 h. Inhibition zone diameters, including the outer diameter of the Oxford cup, were measured with a vernier caliper. Each inhibition zone was measured twice in perpendicular directions, and the mean of the two measurements was recorded. Three parallel plates were used per condition, and the experiments were independently repeated three times. Results are reported as mean ± standard deviation (SD).

#### 3.5.2. Determination of Minimum Inhibitory Concentration (MIC) and Growth Curves by the Flask Macrobroth Dilution Method

Bacterial suspensions were prepared as described in Section 3.5.1. Each activated suspension was adjusted to a 0.5 McFarland standard and then diluted 200-fold with M-H broth to yield a working inoculum of approximately 7.5 × 10^5^ CFU/mL. *Zanthoxylum bungeanum* alkaloid (70% ethanol eluate fraction) was serially diluted twofold in M-H broth. Final concentrations were: 0, 0.5, 1, 2, 4, 8, and 16 mg/mL for *S. aureus* and *B. subtilis*; 0, 0.25, 0.5, 1, 2, 4, 8, and 16 mg/mL for *E. coli*; and 0, 1, 2, 4, 8, 16, and 32 mg/mL for *P. aeruginosa*. DMSO was included as a co-solvent in all alkaloid solutions. In sterile 50 mL flasks, 5 mL of a twofold concentrated alkaloid solution (70% ethanol eluate fraction) was combined with 5 mL of the working bacterial suspension, yielding a final volume of 10 mL per flask. Each concentration was tested in triplicate. A positive control (5 mL M-H broth plus 5 mL bacterial suspension, without alkaloid) and a negative control (10 mL M-H broth, without bacteria) were included. Flasks were incubated at 37 °C with shaking at 180 rpm for 24 h. Samples were collected at 0, 4, 8, 12, 16, 20, and 24 h. Before each sampling, flasks were gently swirled to homogenize the contents, and 200 μL of suspension was transferred to a 96-well plate. Each flask was sampled in triplicate as technical replicates. Optical density at 600 nm (OD_600_) was measured with a microplate reader. Because the alkaloid solution was intrinsically colored and interfered with OD_600_ at higher concentrations, OD_600_ at each time point was corrected by subtracting the background OD_600_ at 0 h for the same concentration: corrected OD_600_(t) = OD_600_(t) − OD_600_(0). Growth curves were plotted with time on the abscissa and corrected OD_600_ on the ordinate. The MIC was defined as the lowest concentration that fully inhibited bacterial growth, using the criteria: corrected OD_600_ ≤ 0.05 and visually clear wells without turbidity.

#### 3.5.3. Determination of Minimum Bactericidal Concentration (MBC)

The MBC was determined according to CLSI M26-A (1999) [34]. After the 24 h incubation described in Section 3.5.2, 1 mL aliquots were taken from the positive control (0 mg/mL), the MIC well, and the wells at one and two concentrations above the MIC. Each aliquot was transferred to a sterile Eppendorf tube and serially diluted 10-fold in sterile physiological saline. For plating, the positive control suspension (0 mg/mL) was diluted to 10^−5^ and 20 μL was spread evenly onto M-H agar plates, while the treated-group suspensions were diluted to 10^−3^ and 20 μL was plated. Each dilution was plated in triplicate. Plates were incubated inverted at 37 °C for 24 h, and colonies were counted on plates containing 30–300 CFU. The initial inoculum was calculated from the colony counts of the positive control at the 10^−5^ dilution using the formula initial inoculum (CFU/mL) = colony count × 10^5^ × (1000/20) = colony count × 5 × 10^6^. Surviving cell concentrations in treated groups were calculated from colony counts at the 10^−3^ dilution using the formula surviving cells (CFU/mL) = colony count × 10^3^ × 50 = colony count × 5 × 10^4^. The MBC was defined as the lowest concentration that reduced the surviving cell count by ≥99.9% relative to the initial inoculum, corresponding to surviving cells ≤ 0.1% of the initial inoculum. If no colonies grew on a plate, the colony count was recorded as 0 CFU/plate. Bactericidal versus bacteriostatic activity was classified by the MBC/MIC ratio: an MBC/MIC ratio ≤ 4 indicated bactericidal activity, whereas a ratio > 4 indicated bacteriostatic activity. If no MBC was detected within the tested concentration range, the agent was classified as bacteriostatic. Each experiment used three parallel samples and was repeated independently three times; results are reported as mean ± standard deviation.

### 3.6. Statistical Analysis

All experiments were performed independently three times, with three parallel replicates per concentration in each experiment. Data are reported as mean ± SD. Growth curves were generated using Origin. Statistical analyses of inhibition zone diameters and MIC/MBC values were conducted with GraphPad Prism version 9.0 and SPSS PRO version 1.0.11.

## 4. Conclusions

This study provides comprehensive process and activity data to support the development of *Zanthoxylum bungeanum* alkaloids as natural products. A stable and efficient extraction process was established and validated at scale. For purification, NKA-9 was selected as the optimal resin from among three candidates, with 70% ethanol serving as the most effective elution solvent. Bioactivity assessments confirmed the in vitro antioxidant potential of the 70% ethanol eluate fraction. Although less potent than ascorbic acid (IC_50_ ≈ 0.0057 mg/mL for DPPH vs. 0.013 mg/mL for the 70% ethanol eluate fraction), the 70% ethanol eluate fraction exhibited a clear dose-dependent scavenging response across all three antioxidant assays. Antibacterial results demonstrated inhibitory effects against both Gram-negative (*E. coli*, *P. aeruginosa*) and Gram-positive (*B. subtilis*, *S. aureus*) bacteria, with strain-specific potency. The most potent activity was observed against *E. coli*, with a minimum inhibitory concentration (MIC) of 2 mg/mL and a minimum bactericidal concentration (MBC) of 8 mg/mL (MBC/MIC = 4), indicating bactericidal activity. In contrast, the activity against *S. aureus* and *P. aeruginosa* was primarily bacteriostatic within the tested concentration range. In summary, beyond process optimization, this work substantiates through detailed activity data the clear potential of *Zanthoxylum bungeanum* alkaloids for development as functional food antioxidants or natural antibacterial agents targeting specific pathogens.

## Figures and Tables

**Table 1:** Results of orthogonal experiment.

No.	A	B	C	D	Total Alkaloid Yield (%)
1	1	1	1	1	0.308
2	1	2	2	2	0.329
3	1	3	3	3	0.339
4	2	1	2	3	0.359
5	2	2	3	1	0.391
6	2	3	1	2	0.334
7	3	1	3	2	0.365
8	3	2	1	3	0.401
9	3	3	2	1	0.431
K1	0.976	1.032	1.043	1.13	
K2	1.084	1.121	1.119	1.028	
K3	1.197	1.104	1.095	1.099	
$\overset{-}{K}$ 1	0.325	0.344	0.348	0.377	
$\overset{-}{K}$ 2	0.361	0.374	0.373	0.343	
$\overset{-}{K}$ 3	0.399	0.368	0.365	0.366	
R	0.074	0.03	0.025	0.034	
Number of Levels	3	3	3	3	
Replicates per Level	3	3	3	3	
Optimal Leve	3	2	2	1	
Factor Priority	A > D > B > C				

Note: A: Particle size (Mesh), B: Ethanol concentration (%), C: Solid-to-liquid ratio (g/mL), D: pH.

**Table 2:** Analysis of Variance.

	Sum of Squares	df	Mean Square	F-Ratio	*p*
Intercept	1.179	1	1.179	2342.767	0.000 **
A	0.008	2	0.004	8.091	0.110
B	0.001	2	0.001	1.479	0.403
D	0.002	2	0.001	1.812	0.356
Residual	0.001	2	0.001		

Note: R^2^ = 0.919; ** *p* < 0.01.

**Table 3:** Verification of the optimized extraction process.

Category	Details	Value
Optimal Levels	A_3_B_2_C_2_D_1_	40/65%/1:15/2
Validation Data	Yield 1/Yield 2/Yield 3	0.463/0.466/0.466
Statistics	Mean ± SD	0.465 ± 0.006%
Quality Control	RSD	<1.5%
Comparison	vs. A_3_B_3_C_2_D_1_	7.9%

**Table 4:** Comparison of resin adsorption capacity.

Resin Type	Static Adsorption Capacity (mg/g)	Static Adsorption Rate	Desorption Amount (mg/g)	Static Desorption Rate
D101	13.27	55.43%	9.24	69.63%
AB-8	11.93	49.83%	7.39	61.94%
NKA-9	14.89	62.20%	10.82	72.67%

**Table 5:** Recovery yield (%) of alkaloids from different macroporous resins using gradient ethanol elution.

Resin Type	Alkaloid Content%		
	D101	AB-8	NKA-9
Eluent: ethanol (50%)	0.98	0.75	0.86
Eluent: ethanol (70%)	5.68	4.24	5.97
Eluent: ethanol (90%)	1.24	1.68	1.12

**Table 6:** Dry weight of samples eluted with varying ethanol concentrations.

Elution Solvent Concentration	Dry Sample Weight (g)
50% ethanol	1.941
70% ethanol	0.909
90% ethanol	1.717

**Table 7:** Regression parameters and half-maximal inhibitory concentrations (IC_50_) for the DPPH· scavenging activity of *Zanthoxylum bungeanum* alkaloid extracts.

Sample	Linear Equations	IC_50_ (mg/mL)
crude extract	y = 3.873x + 0.4443, R^2^ = 0.982	0.014
70% ethanol eluate fraction	y = 5.7713x + 0.4212, R^2^ = 0.980	0.013
VC (Positive Control)	y = 92.939x − 0.0294, R^2^ = 0.997	0.0057

Note: In the linear equation, y is the clearance rate (%), and x is the sample concentration (mg/mL).

**Table 8:** Regression parameters and half-maximal inhibitory concentrations (IC_50_) for the ABTS^+^· scavenging activity of *Zanthoxylum bungeanum* alkaloid extracts.

Sample	Linear Equations	IC_50_ (mg/mL)
crude extract	y = 6.7092x + 0.0592, R^2^ = 0.9974	0.066
70% ethanol eluate fraction	y = 7.1362x + 0.0504, R^2^ = 0.9712	0.063
VC (Positive Control)	y = 11.633x − 0.124, R^2^ = 0.9871	0.054

Note: In the linear equation, y is the clearance rate (%), and x is the sample concentration (mg/mL).

**Table 9:** Regression parameters and half-effect concentrations (EC_50_) for the Fe^3+^-reduction activity of *Zanthoxylum bungeanum* alkaloid extracts.

Sample	Linear Equations	EC_50_ (mg/mL)
crude extract	y = 3.0744x + 0.0855, R^2^ = 0.9783	0.135
70% ethanol eluate fraction	y = 3.9232x − 0.0156, R^2^ = 0.9482	0.131
VC (Positive Control)	y = 15.195x + 0.029, R^2^ = 0.9569	0.031

Note: In the linear equation, y is the clearance rate (%), and x is the sample concentration (mg/mL).

**Table 10:** Inhibition zone diameters (mm) of *Zanthoxylum bungeanum* alkaloids (10 mg/mL) against four bacterial strains (mean ± SD).

Test Strains	Antibacterial Diameter (mm)
(A) *Escherichia coli* (*E. coli*)	20.27 ± 0.25
(B) *Bacillus subtilis* (*B. subtilis*)	17.27 ± 0.16
(C) *Staphylococcus aureus* (*S. aureus*)	16.67 ± 0.20
(D) *Pseudomonas aeruginosa* (*P. aeruginosa*)	15.93 ± 0.17

Note: The Oxford cup has an outer diameter of 6 mm, which is included in the reported diameters. The solvent control (DMSO) showed no inhibition zone.

**Table 11:** Minimum bactericidal concentrations (MBC) of *Zanthoxylum bungeanum* alkaloids against tested bacterial strains.

Test Strain	Conc. (mg/mL)	Colony Counts (CFU/Plate, Mean ± SD)	MBC (mg/mL)	MBC/MIC	Type
*E. coli* (MIC = 2)	0	66.7 ± 6.1	–	–	
	1	258.3 ± 72.4	–	–	
	2	110.0 ± 23.6	–	–	
	4	44.7 ± 17.0	–	–	
	8	3.0 ± 2.6	8	4	Bactericidal
*B. subtilis* (MIC = 4)	0	98.7 ± 13.2	–	–	
	2	498.7 ± 83.5	–	–	
	4	178.3 ± 40.8	–	–	
	8	69.7 ± 21.1	–	–	
	16	7.3 ± 3.2	16	4	Bactericidal
*S. aureus* (MIC = 4)	0	115.3 ± 21.0	–	–	
	2	1039.0 ± 68.3	–	–	
	4	746.3 ± 126.1	–	–	
	8	650.0 ± 136.6	–	–	
	16	439.0 ± 70.1	ND	>4	Bacteriostatic
*P. aeruginosa* (MIC = 8)	0	105.0 ± 16.1	–	–	
	4	1020.7 ± 115.8	–	–	
	8	954.3 ± 100.0	–	–	
	16	778.7 ± 151.4	–	–	
	32	773.3 ± 103.8	ND	>4	Bacteriostatic

Note: For the positive control (0 mg/mL), 10^−5^ diluted suspension was plated (20 μL/plate). For treated groups, 10^−3^ diluted suspension was plated (20 μL/plate). Initial inocula: *E. coli* 3.34 × 10^8^, *B. subtilis* 4.94 × 10^8^, *S. aureus* 5.77 × 10^8^, *P. aeruginosa* 5.25 × 10^8^ CFU/mL. The 99.9% killing thresholds (CFU/plate at 10^−3^ dilution) are 6.68, 9.88, 11.54, and 10.50, respectively. ND: not detected. MBC/MIC ≤ 4: bactericidal; MBC/MIC > 4: bacteriostatic.

**Table 12:** Component analysis of the 70% ethanol eluted fraction in positive ion mode ([M + H]^+^).

NO.	Retention Time (t/min)	Compound	Molecular Formula	Adduct	Theoretical Value (*m*/*z*)	Measured Value (*m*/*z*)	Error (ppm)	Fragment Ion (*m*/*z*)
1	16.49	Timuramide A	C16H25NO4	[M + H]^+^	295.1784	296.1863	−2.3	296.1860, 147.0805, 121.0647, 91.0542
2	16.91	Timuramide A (isomeric)	C16H25NO4	[M + H]^+^	295.1784	296.1862	−2.2	296.1861, 121.0649, 147.0805, 91.0548
3	34.13	Hydroxy-ε-sanshool (isomeric)	C16H25NO2	[M + H]^+^	263.1885	264.1965	−2.5	264.1965, 175.1117, 107.0851, 79.0543, 246.1854, 147.1170
4	34.41	Hydroxy-α-sanshool (isomeric)	C16H25NO2	[M + H]^+^	263.1885	264.1966	−3.0	264.1965, 246.1854, 175.1119, 147.1169, 79.0542
5	34.91	Hydroxy-β-sanshool (isomeric)	C16H25NO2	[M + H]^+^	263.1885	264.1968	−3.4	264.1965, 246.1861, 175.1117, 147.1164, 79.0543
6	35.24	α-Sanshool	C16H25NO	[M + H]^+^	247.1936	248.2018	−3.9	248.2012, 79.0543
7	35.64	Hydroxy-γ-sanshool	C18H27NO2	[M + H]^+^	289.2042	290.2126	−4.0	290.2125, 272.2013, 165.1152, 131.0857, 79.0541
8	36.06	Hydroxy-γ-isosanshool	C18H27NO2	[M + H]^+^	289.2042	290.2125	−3.8	290.2125, 272.1993, 165.1150, 131.0858, 79.0543
9	36.20	γ-Sanshool	C18H27NO	[M + H]^+^	273.2093	274.2176	−4.0	274.2171, 105.0700, 91.0543, 79.0542
10	36.31	bungeanool	C18H29NO2	[M + H]^+^	291.2198	292.2282	−4.0	292.2278, 274.2171, 203.1425, 72.0811
11	37.76	Tetrahydro-sanshool	C18H33NO2	[M + H]^+^	295.2511	296.2594	−3.0	296.2593, 278.2485

**Table 13:** Factor level settings for orthogonal experiment.

Level	A Particle Size (Mesh)	B Ethanol Concentration/%	C Solid-to-Liquid Ratio (g/mL)	D pH
1	20	50	1:10	2
2	30	65	1:15	3
3	40	80	1:20	4

## Data Availability

The original contributions presented in this study are included in the article. Further inquiries can be directed to the first author.

## Supplementary Materials

The following supporting information can be downloaded at: https://www.mdpi.com/article/10.3390/molecules31132328/s1, Figure S1: Base Peak Intensity (BPI) chromatograms of the *Zanthoxylum bungeanum* crude extract analyzed by UPLC-HRMS in positive ion mode; Table S1: Gradient Elution Program; Table S2: Gradient Elution Program; Table S3: Component analysis of the *Zanthoxylum bungeanum* crude extract in positive ion mode ([M + H]^+^).
