# Correction: Huang et al. Purification of Alkaloids from *Zanthoxylum bungeanum* Using Macroporous Adsorption Resin and Evaluation of Their Biological Activities. *Molecules* 2026, *31*, 2328

**DOI:** 10.3390/molecules31183149

**Published:** 2026-09-08

**Authors:** Dongdong Huang, Tianyu Zhao, Bohao Wang, Benqun Yang, Zhifeng Li

**Affiliations:** 1 College of Chemical Engineering and Technology, Tianshui Normal University, Tianshui 741001, China; ddhtsnu@126.com (D.H.); zhaoty08@126.com (T.Z.); 15294360059@163.com (B.W.); 2 Key Laboratory of Advanced Optoelectronic Functional Materials of Gansu Province, Tianshui Normal University, Tianshui 741001, China; 3 Key Laboratory for New Molecule Materials Design and Function of Gansu Universities, Tianshui Normal University, Tianshui 741001, China

## Text Correction

In the original publication [1], there was an error in Section 3.3.3. (Purification of Crude Extract Using Macroporous Resin), third paragraph, second sentence.

The sentence “Then, 30 mL of crude extract (prepared by dissolving 7.5 g of dried crude extract in 500 mL, i.e., 15 mg/mL) was added, …” should be corrected to “Then, 15 mL of crude extract (prepared by dissolving 0.75 g of dried crude extract in 500 mL, i.e., 1.5 mg/mL) was added, …”

It should be clarified that the correction of the crude extract concentration from 15 mg/mL to 1.5 mg/mL does not affect the pharmacological evaluation results and conclusions of this study. The static adsorption and desorption experiments described in Section 3.3.3 were performed solely for the screening of macroporous resin types, and the resulting optimal resin as well as the relative adsorption/desorption rates remain valid. The pharmacological activities of the purified alkaloids reported in the subsequent sections were assessed using independently prepared samples and are therefore not impacted by this numerical correction.

The authors state that the scientific conclusions are unaffected. This correction was approved by the Academic Editor. The original publication has also been updated.
