# Supplementary material for: Purification of Alkaloids from Zanthoxylum bungeanum Using Macroporous Adsorption Resin and Evaluation of Their Biological Activities
Source: Molecules. 2026 Jul 2;31(13):2328. doi: 10.3390/molecules31132328 (PMC13362608; doi:10.3390/molecules31132328)
Supplement: Supplementary file 1 [file molecules-31-02328-s001.zip › molecules-4256697-supplementary.pdf]

Supplementary Information

Purification of Alkaloids from Zanthoxylum bungeanum Using Macroporous Adsorption Resin and Evaluation of Their Biological Activities

1. Chromatographic Conditions

The analysis of the analytes was performed on a Waters 1525 Binary HPLC system, equipped with a Waters 1525 binary pump, a column oven, a Waters 2489 UV/Visible detector (UV), and the corresponding workstation software. The chromatograms were recorded at a wavelength of 280 nm. Separation was carried out on a SinoChrom ODS-BP column (4.6 mm × 250 mm; 5 μm) at a column temperature of 35 °C. The mobile phase consisted of 0.2% acetic acid solution (A) and acetonitrile (B), using a gradient elution program (**Table S1**) at a flow rate of 1.0 mL/min, with an injection volume of 20 μL.

Table S1. Gradient Elution Program.

Time (min)	Flow Rate (mL/min)）	Flow A (%)	Flow B (%)
0–15	1	92–75	8–25
15–30	1	75–25	25–75
30–45	1	25–10	75–90
45–50	1	10–92	90–8

1.1. Sample Preparation

Accurately weigh 200 mg of Zanthoxylum crude extract (or 70% ethanol elution fraction) into a stoppered conical flask. Add 10 mL of 50% methanol, sonicate for 20 min (power: 280 W, frequency: 53 kHz), cool to room temperature, and weigh again. Replenish the lost weight with solvent, mix well, filter, and pass through a 0.42 μm organic membrane. Take the successive filtrate for analysis.

2. UPLC-IM-QTOF/MS Analysis Conditions

2.1. Liquid Chromatography Conditions

Liquid chromatography separation was performed on an Agilent 1260 UHPLC system equipped with a quaternary pump. A Waters ACQUITY UPLC HSS T3 column (1.8 μm, 2.1 mm × 100 mm) was employed. The column temperature was maintained at 30 °C. The mobile phase consisted of acetonitrile (A) and 0.1% formic acid in water (B). The flow rate was set at 0.3 mL/min, and the injection volume was 3 μL. The gradient elution program is detailed in Table S2.

Table S2. Gradient Elution Program.

Time (min)	Flow Rate (mL/min)）	Flow A(%)	Flow B(%)
0	0.3	10	95
18	0.3	35	65
24	0.3	46	54
30	0.3	90	10
32	0.3	100	0
35	0.3	100	0
36	0.3	10	90
40	0.3	10	90

2.2. Sample Preparation

Accurately weigh 50 mg of Zanthoxylum crude extract (or 70% ethanol elution fraction) into a stoppered conical flask. Add 10 mL of 50% methanol, sonicate for 20 min (power: 280 W, frequency: 53 kHz), cool to room temperature, and weigh again. Replenish the lost weight with solvent, mix well, filter, centrifuge at 12 000 r/min for 15 min, and pass through a 0.22 μm organic membrane. Take the successive filtrate for analysis.

2.3. Mass Spectrometry Conditions

Mass spectrometric detection was performed using an electrospray ionization (ESI) source in positive ion mode. The capillary voltage was set at 3500 V, the source temperature at 225 °C, the drying gas flow rate at 7 L/min, the nebulizer pressure at 25 psi, the sheath gas temperature at 250 °C, the sheath gas flow rate at 12 L/min, and the fragmentor voltage at 400 V. The mass-to-charge (m/z) acquisition range was set from 50 to 1500 Da. The collision energy was set to 10 V. Data were acquired in automatic data-dependent acquisition (DDA) mode with an MS/MS trigger threshold of 500 counts. Data were qualitatively analyzed using Agilent MassHunter Workstation Software (Version B.08.00).

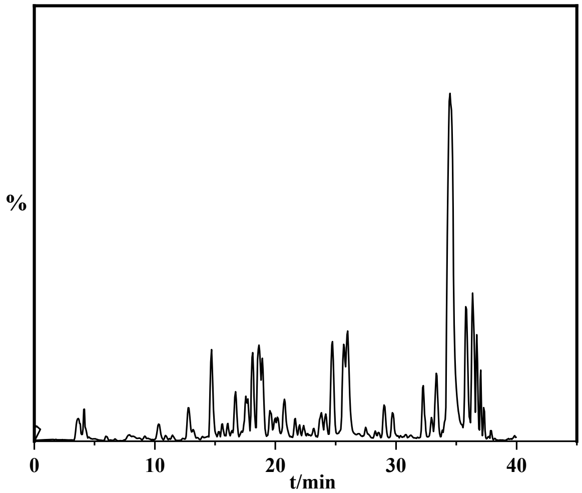

Figure S1. Base Peak Intensity (BPI) chromatograms of the crude extract analyzed by UPLC-HRMS in positive ion mode.

Table S3. Component analysis of the Zanthoxylum bungeanum Crude Extract in positive ion mode ([M+H]⁺.

NO.	Retention Time（t/min）	Compound	Molecular Formula	Adduct	Theoretical Value(m/z)	Measured Value（m/z）	Error (ppm)	Fragment Ion (m/z)
1	6.19	γ-Fagarine	C13H12NO3	$[M+Na]^+$	230.0817	253.0708	-0.1	253.0708
2	14.88	Magnoflorine	C20H24NO4+	$[M]^+$	342.1705	342.1706	-0.3	342.1705、297.1125、282.0898、265.0857、237.0910、191.0859、222.0647、165.0701
3	16.55	Timuramide A	C16 H25 N O4	$[M+H]^+$	295.1784	296.1861	-2.2	296.1860、147.0806、121.0648、91.0541
4	16.90	Timuramide A（isomeric）	C16 H25 N O4	$[M+H]^+$	295.1784	296.1861	-2.1	296.1860、121.0644、91.0547
5	17.68	Tetrahydropalmatine	C21 H25 N O4	$[M+H]^+$	355.1784	356.1868	-3.1	356.1859
6	34.18	Hydroxy-ε-sanshool（isomeric）	C16 H25 N O2	$[M+H]^+$	263.1885	264.1964	-2.5	264.1958、246.1852、147.1169、175.1122、79.0541
7	34.91	Hydroxy-α-sanshool（isomeric）	C16 H25 N O2	$[M+H]^+$	263.1885	264.1968	-3.6	264.1967、246.1852、175.1117、147.1170、79.0543
8	35.06	Hydroxy-β-sanshool（isomeric）	C16 H25 N O2	$[M+H]^+$	263.1885	264.1967	-3.4	264.1974、246.1856、175.1122、147.1171、79.0544
9	35.26	α-Sanshool	C16 H25 N O	$[M+H]^+$	247.1936	248.2017	-3.2	248.2016、175.1119、79.0543、
10	35.66	Hydroxy-γ-sanshool	C18 H27 N O2	$[M+H]^+$	289.2042	290.2127	-4.3	290.2121、272.2014、165.1151、79.0542、131.0860
11	36.05	Hydroxy-γ-isosanshool	C18 H27 N O2	$[M+H]^+$	289.2042	290.2125	-3.4	290.2125、272.2010、165、1151、131.0861、79.0543
12	36.21	γ-Sanshool	C18 H27 N O	$[M+H]^+$	273.2093	274.2175	-3.5	274.2171、105.0696、91.0541、79.0541
13	36.33	bungeanool	C18 H29 N O2	$[M+H]^+$	291.2198	292.2280	-3.3	292.2275、274.2173、203.1426、72.0809
14	37.85	Tetrahydroxy-sanshool	C18 H33 N O2	$[M+H]^+$	295.2511	296.2592	-2.3	296.2590、278.2484
